# Supplementary material for: Network pharmacological insight into traditional bone healing practices of Sikkim, India
Source: PLoS One. 2026 Apr 15;21(4):e0346125. doi: 10.1371/journal.pone.0346125 (PMC13082723; doi:10.1371/journal.pone.0346125)
Supplement: S1 File — Methodology of ethnopharmacological survey and documentation with attached questionnaires. (DOCX) [file pone.0346125.s001.docx]

**Network Pharmacological Insight into Traditional Bone Healing Practices of Sikkim, India**

Mukunda Anuj Sharma^1,2^, Bharat Gopalrao Somkuwar^3, 22^, Parvin A Barbhuiya^1^, Bhumika Gurung^1^, Madhusmita Mahapatra^1^, Firdous Fatima^3^, Teresa Ningthoujam^1, 22^, Ashika Bhattarai^1^, Bikash Rai^1^, Pravin Kumar^1^, Bishal Tiwari^1^, Sancha Kumar Subba^4^, Zyankit Lepcha^5^, Purna Maya Gurung^6^, Nandalal Khadka^7^, Ratan Bahadur Tamang^8^, Balbir Khati^9^, Shribhakta Chettri^10^, Ran Bahadur Rai^11^, Hem Lall Sharma^12^, Yam Bahadur Rai^13^, Norzang Lepcha^14^, Theptuk Lepcha^15^, Pritiman Singh Chettri^16^, Monraj Limboo^17^, Theweng Gyenthen^18^, Tulshi Pradhan^19^, Prem Gurung^20^, Prem Prashad Dhakal^21^**,** Nanaocha Sharma^1, 22^**,** Lokesh Deb^1, 22*^

1. Biotechnology Research and Innovation Council - Institute of Bioresources and Sustainable Development (BRIC-IBSD) - Regional Centre, Sikkim (Department of Biotechnology, Government of India), 5th Mile, NH-10A, Near Metro Point, Tadong, Gangtok, Sikkim -795001, India.
2. Department of Zoology, Sikkim University, 6^th^ Mile, Tadong, Gangtok, Sikkim, India.
3. Biotechnology Research and Innovation Council-Institute of Bioresources and Sustainable Development - Mizoram (Department of Biotechnology, Government of India), A-1, Nursery Veng, Chawanga Road, Aizawl-796005, India.
4. Traditional Healer, Pachey Village, Samsing, Pakyong, Sikkim – 737106, India.
5. Traditional Healer, Lower Radhu, Dentam, Geyzing, Sikkim, Sikkim - 737113, India.
6. Traditional Healer, Upper Chuba, Phongla, Namchi, Sikkim -737126, India
7. Traditional Healer, Tangzi, Rateypani, Namchi, Sikkim-737126, India
8. Traditional Healer, Dhaje Dhara, Nazi Ruchung, Namchi, Sikkim- 737126, India
9. Traditional Healer, Lower Rangang, Yangyang, Namchi, Sikkim -737134, India
10. Traditional Healer, Namphok, Yangyang, Namchi, Sikkim -737126, India
11. Traditional Healer, Ladam Machong, Pakyong, Sikkim -737131, India
12. Traditional Healer, Assam Daragong, Assam Linzey, Pakyong, Sikkim -737135, India
13. Traditional Healer, Titiribotey, Rorathang, Pakyong, Sikkim 737133, India
14. Traditional Healer, Shipgyer, Mangan, Sikkim -737116, India
15. Traditional Healer, Upper Gor, Lower Dzongu, Mangan, Sikkim -737116, India
16. Traditional Healer, Upper Singhik, Mangan, Near MSSS, Mangan, Sikkim -737116, India
17. Traditional Healer, Mangshila, Upper Ralak, Mangan, Sikkim – 737116, India
18. Traditional Healer, Shakathang, Lachung, Katao Road, Mangan, Sikkim -737120, India
19. Traditional Healer, Lower Timburbong, Soreng, Sikkim – 737121, India
20. Traditional Healer, Namcheybong, Pakyong, Sikkim – 737106, India
21. Traditional Healer, Aritar, Khamdong Singtam, Gangtok, Sikkim-737134, India.
22. Biotechnology Research and Innovation Council - Institute of Bioresources and Sustainable Development (BRIC-IBSD) (Department of Biotechnology, Government of India), Takyelpat, Imphal, Manipur -795001, India.

**Note:** Bharat Gopalrao Somkuwar and Mukunda Anuj Sharma contributed equally

**Short running title:** Traditional Healthcare Practices of Sikkim

***Corresponding address:**

**Lokesh Deb, M. Pharma, Ph.D.**

**Scientist – E (Pharmacology)**

Biotechnology Research and Innovation Council -Institute of Bioresources and Sustainable Development (BRIC-IBSD)– Regional Centre, Sikkim, (Department of Biotechnology, Government of India), 5^th^ mile, NH-10A, Near Metro Point, Tadong, Gangtok -737102,

Sikkim, India; Mob –+919436890969; Email: [lokeshdeb@gmail.com](mailto:lokeshdeb@gmail.com); [lokeshdeb.ibsd@nic.in](mailto:lokeshdeb.ibsd@nic.in)

IBSD Manuscript No. - IBSD/MS/2020/01/12

**Supplementary materials**

**Methodology for Ethnopharmacological survey and documentation of Traditional healthcare practices of Sikkim**

An exploratory study was conducted to collect data, and an ethnopharmacological survey (a semi-structured, questionnaire-based, cross-sectional, descriptive study) was performed from April 2022 to May 2024 in all six districts of Sikkim (Mangan, Gyalshing, Gangtok, Namchi, Pakyong, and Soreng). Ethnomedicinal data on plants used for bone mending were collected through interviews with 26 respondent healers from different localities. The objective of the study was clearly explained in the local language (Nepali). The participants who were willing to share information provided written consent (on a consent form). All the respondent healers from all six Districts of Sikkim were considered for the study. All participants were interviewed in their regional language, and information was recorded regarding the specific plants they used for bone mending. The data was also collected using a pre-designed questionnaire presented in both written and audio-visual formats. The informants were asked to provide details on the parts of plants used, other non-plant materials incorporated, the mode of preparation, the methods employed (decoction, juice, infusion, or powder), the modes of administration, and the duration of treatment. Transect walks and field observation supplemented by interviews. During the transect walks, plants were carefully observed and collected under the guidance of traditional healers.

**Letter of Consent Form**

(Used for seeking written permission of Traditional Practitioners during the Cross Culture Ethnopharmacological Survey on ‘Traditional Healthcare Practices in Sikkim’ conducting by the Institute of Bioresources and Sustainable Development- Regional Centre, Sikkim, Tadong, Gangtok, Sikkim-737102, India)

**[Translated in respective local language of the survey area]**

**Ref. No.:** IBSD-RCS/2022/EPS/

I, …………………….. residence …………………………………………hereby giving my permission to the Institute of Bioresources and Sustainable Development (IBSD) – Regional Centre, Sikkim (A national Institute of Department of Biotechnology, Government of India), 5^th^ Mile, NH-10A, Tadong, Gangtok-737102, Sikkim, India for using my knowledge of Traditional Health Care Practices/Traditional food for the purpose of research. I do not have any objection for using my Photographs/Audio-visual video/Audio recording captured during the Ethnopharmacological Survey conducted by the research team of IBSD- regional Centre, Sikkim. I have been explaining the matter of this letter by translating in Nepali before giving my signature. I am giving my consent for above cited purpose with my full knowledge and consciousness.

**[Translate in respective local language of the survey area]**

**Signature of** **the Informer**

**Date:**

### ETHNOPHARMACOLOGICAL SURVEY DATA SHEET – I

(Description of the Healer)

| **1.** | **Date of Survey** |  |
| --- | --- | --- |
| **2.** | **Name of Traditional Practitioner** |  |
| **3.** | **Name of Place & Address** |  |
| **4.** | **Age & Sex** |  |
| **5.** | **Community** |  |
| **6.** | **Global Position data (GPS)** | **Longitude: E………………………………..**  **Latitude: N……………………….…………**  **Altitude: ……………………………………ASL** |
| **7.** | **Experience (in year)** |  |
| **8** | **Name of the Health Condition(s) treated by the Traditional Healers** | |
|  | **Local Name** | **Medical term/in English** |
| **A** |  |  |
| **B** |  |  |
| **C** |  |  |
| **D** |  |  |
| **E** |  |  |

**Documented By: Date:**

### ETHNOPHARMACOLOGICAL SURVEY DATA SHEET – II

**(Description of the Health Condition & Treatment Details)**

(Use separate sheets for each treatment)

| **1.** | **Name of Disease Condition**  **(As per traditional Healer)** |  | | |
| --- | --- | --- | --- | --- |
| **2.** | **Affected part of the body/organ** |  | | |
| **3.** | **Symptoms as per the Healer** |  | | |
| **4.** | **Causes/risk factor/Etiology** |  | | |
| **5.** | **Stages/severity of condition** | **Treating Preliminary / Mild Stage / Moderate/severe** | | |
| **6.** | **Remedy/Procedure/Treatment** | | | |
| **Sl. No** | **Local name of ingredients (If plant, tree/herb/shrub/climber)** | **Scientific Name** | **Part used** | **Collection Method & Condition used (Dry/Fresh)** |
| **A** |  |  |  |  |
| **B** |  |  |  |  |
| **C** |  |  |  |  |
| **D** |  |  |  |  |
| **E** |  |  |  |  |
| **7.** | **Method of preparation of traditional medicine in details (including Vehicle/Adjuvant used with quantity, if any)** |  | | |
| **8.** | **Storage method, if any** |  | | |
| **7.** | **Any pre or post treatment procedure?** |  | | |
| **9.** | **Doses (Convert in ml/gm) and Mode of administration (Internal/external)** | **Adult** | | **Child** |
| **10.** | **Duration of Treatment** |  | | |
| **11.** | **Food restriction during treatment** |  | | |

**Documented By: Date:**

**Bioresources Specimen/Herbarium details**

| **Specimen No** | **Local name of the bioresource** | **Botanical/Zoological/ Chemical (Scientific) name** | **Habit** | **Collected from including GPS data** |
| --- | --- | --- | --- | --- |
|  |  |  |  |  |
|  |  |  |  |  |
|  |  |  |  |  |
|  |  |  |  |  |
|  |  |  |  |  |

**Collected by: Date:**

**Identified by: Authenticated by:**
