# Supplementary figures and images for: Network pharmacological insight into traditional bone healing practices of Sikkim, India
Source: PLoS One. 2026 Apr 15;21(4):e0346125. doi: 10.1371/journal.pone.0346125 (PMC13082723; doi:10.1371/journal.pone.0346125)

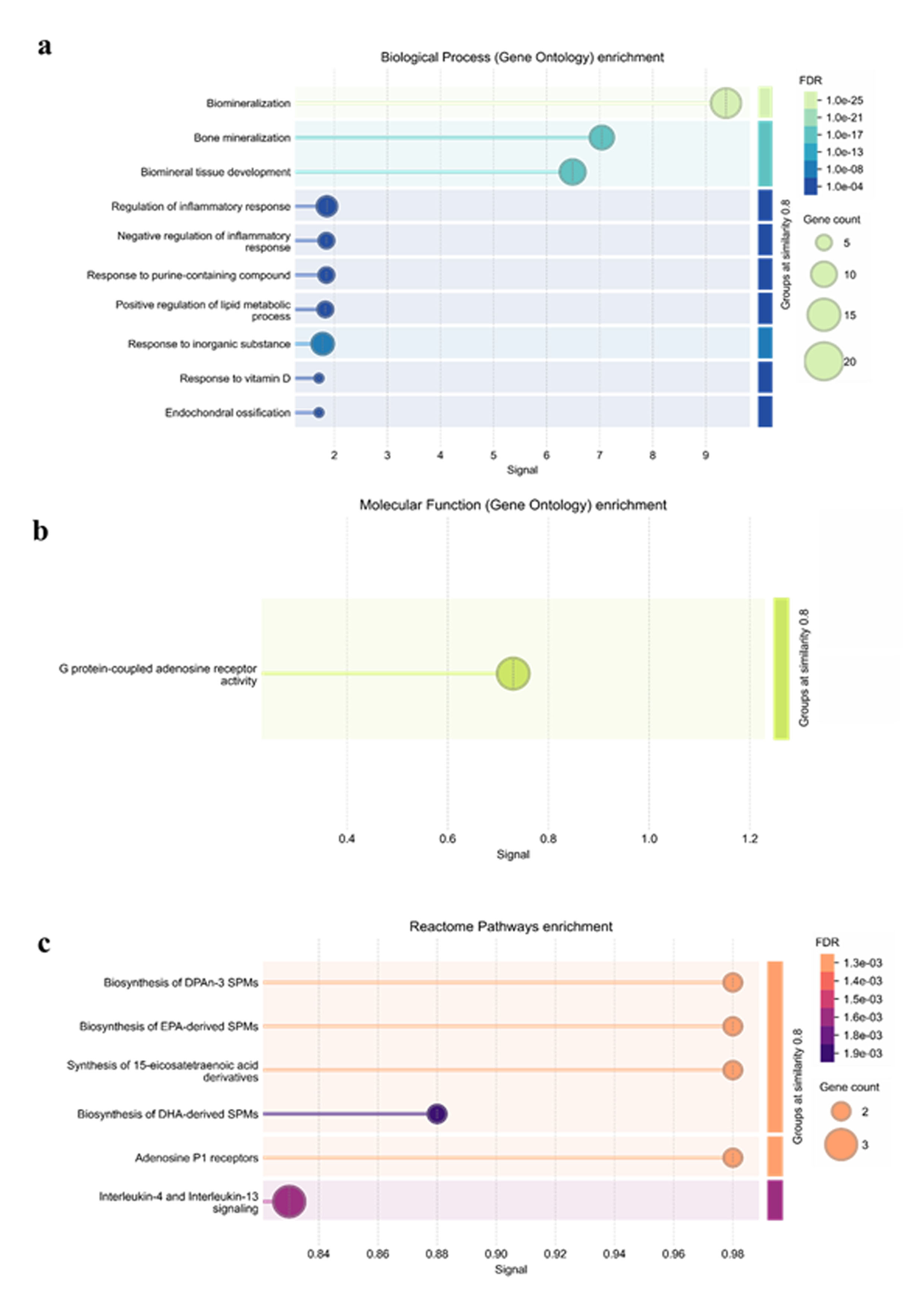

Supplement: S1 Fig — (JPG) [file pone.0346125.s002.jpg]

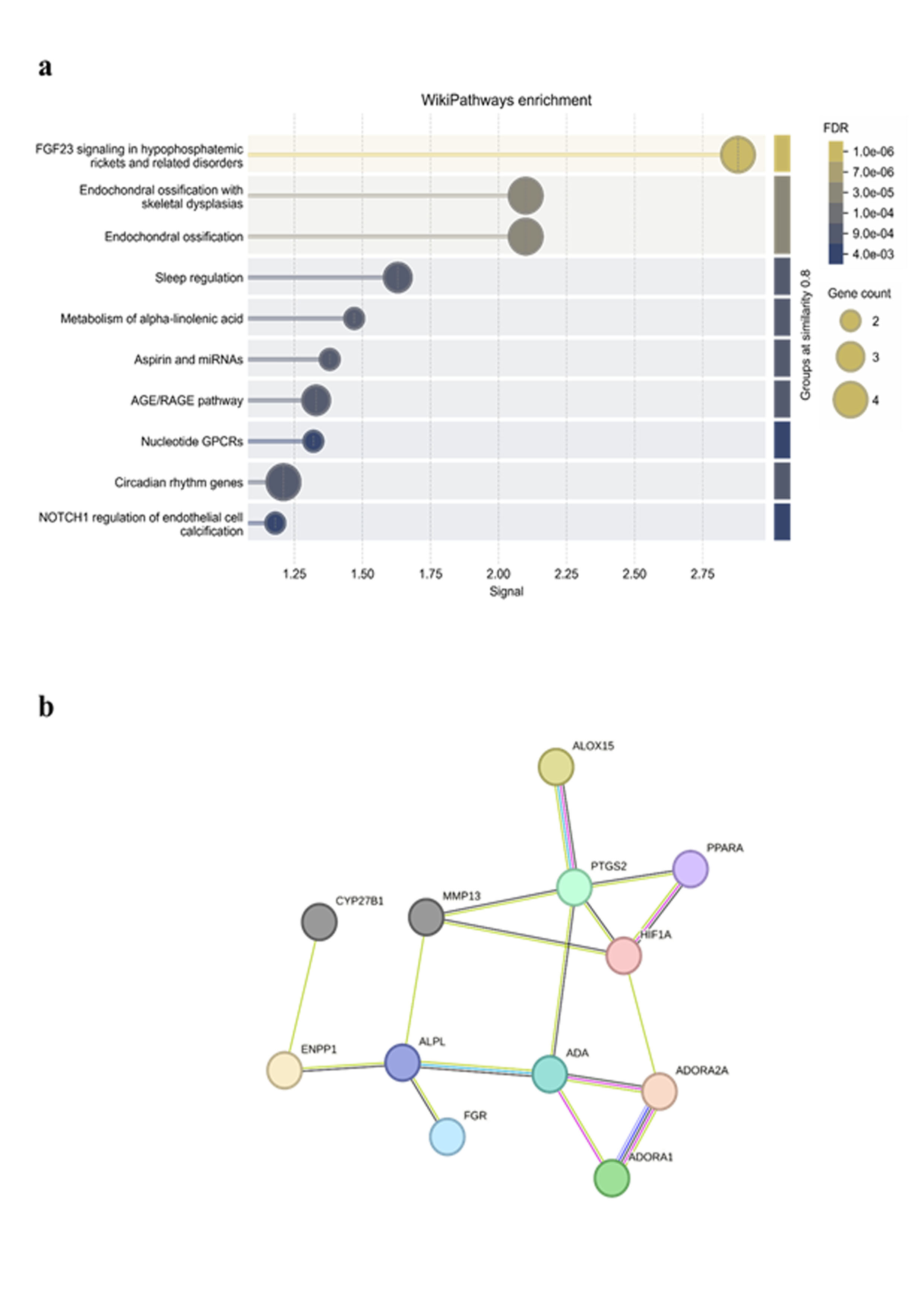

Supplement: S2 Fig — (JPG) [file pone.0346125.s003.jpg]

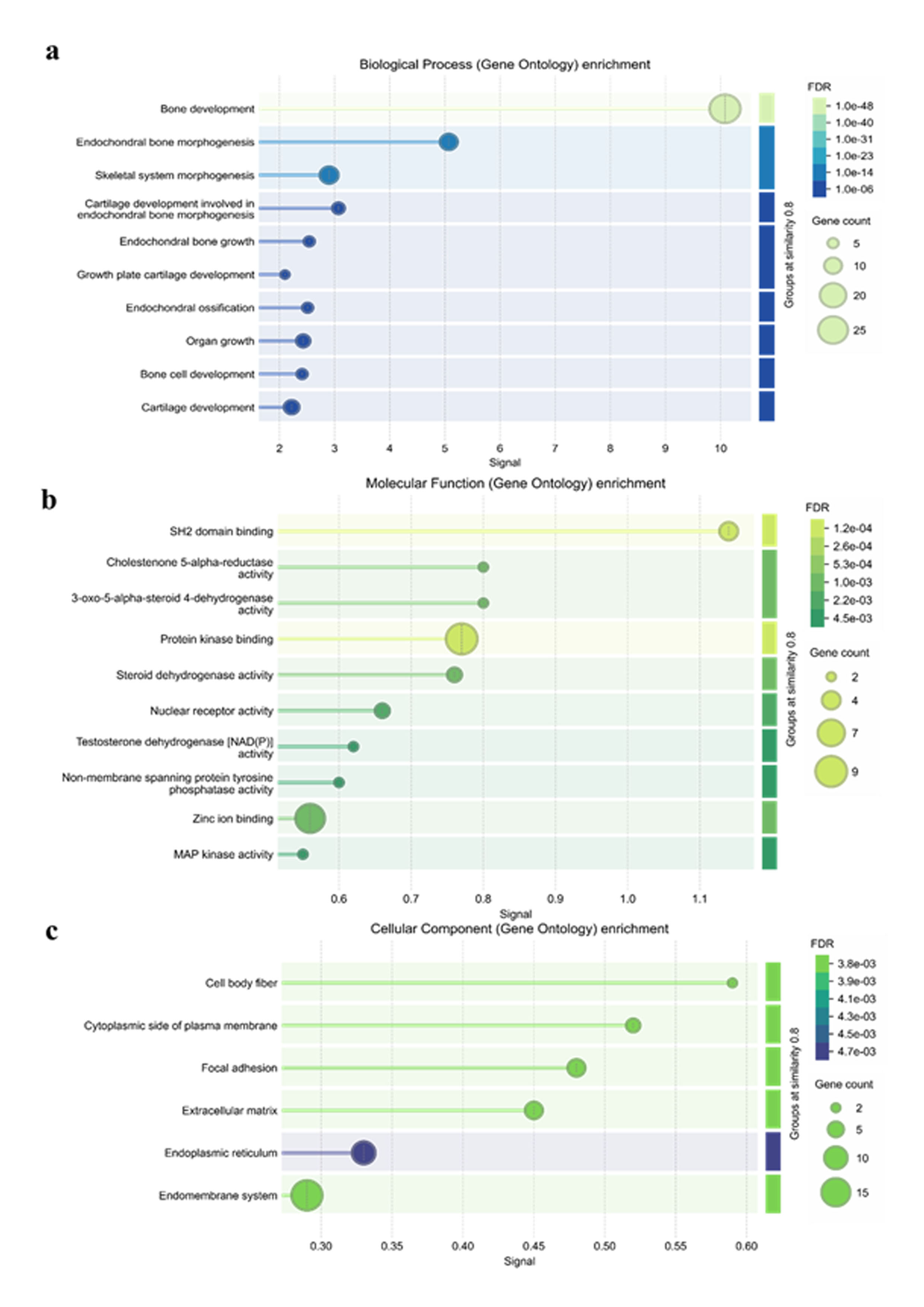

Supplement: S3 Fig — (JPG) [file pone.0346125.s004.jpg]

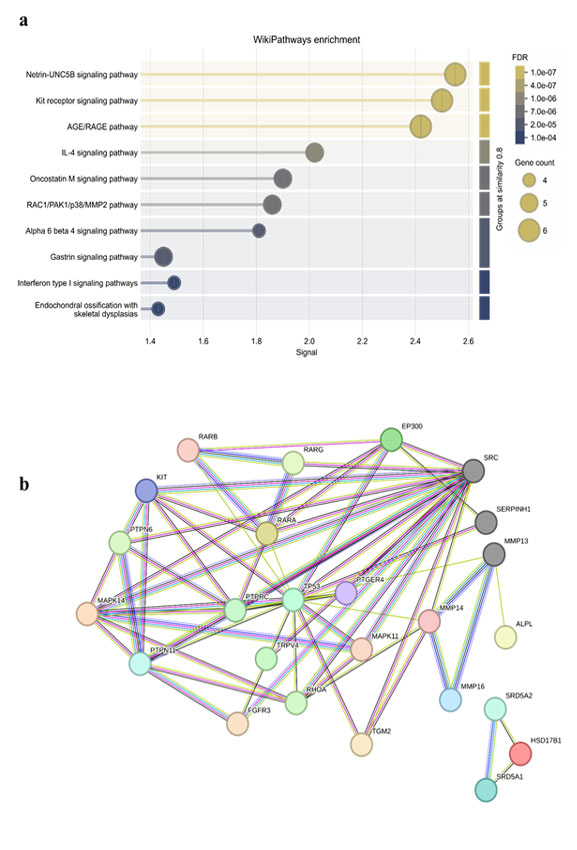

Supplement: S4 Fig — (JPG) [file pone.0346125.s005.jpg]

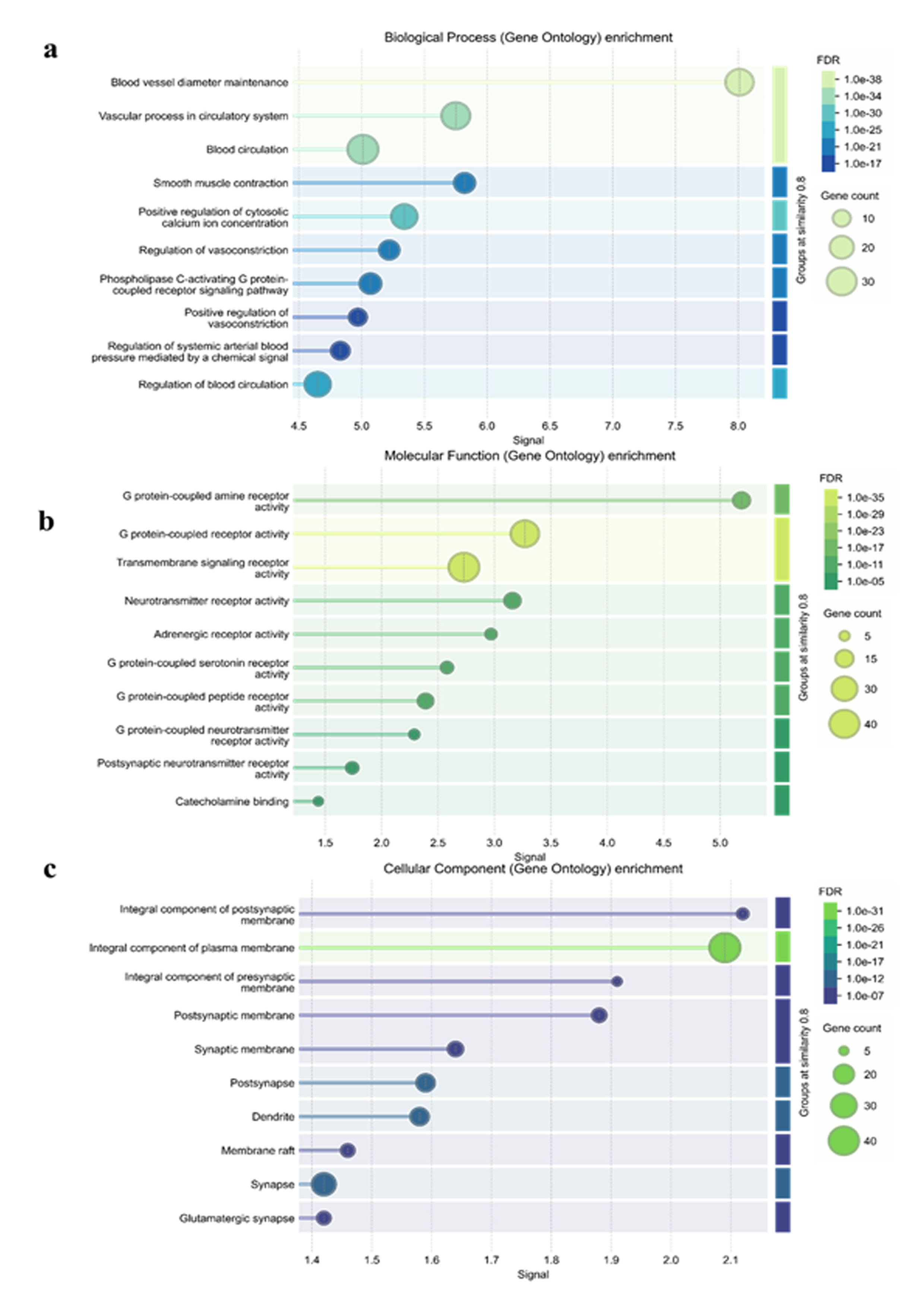

Supplement: S5 Fig — (JPG) [file pone.0346125.s006.jpg]

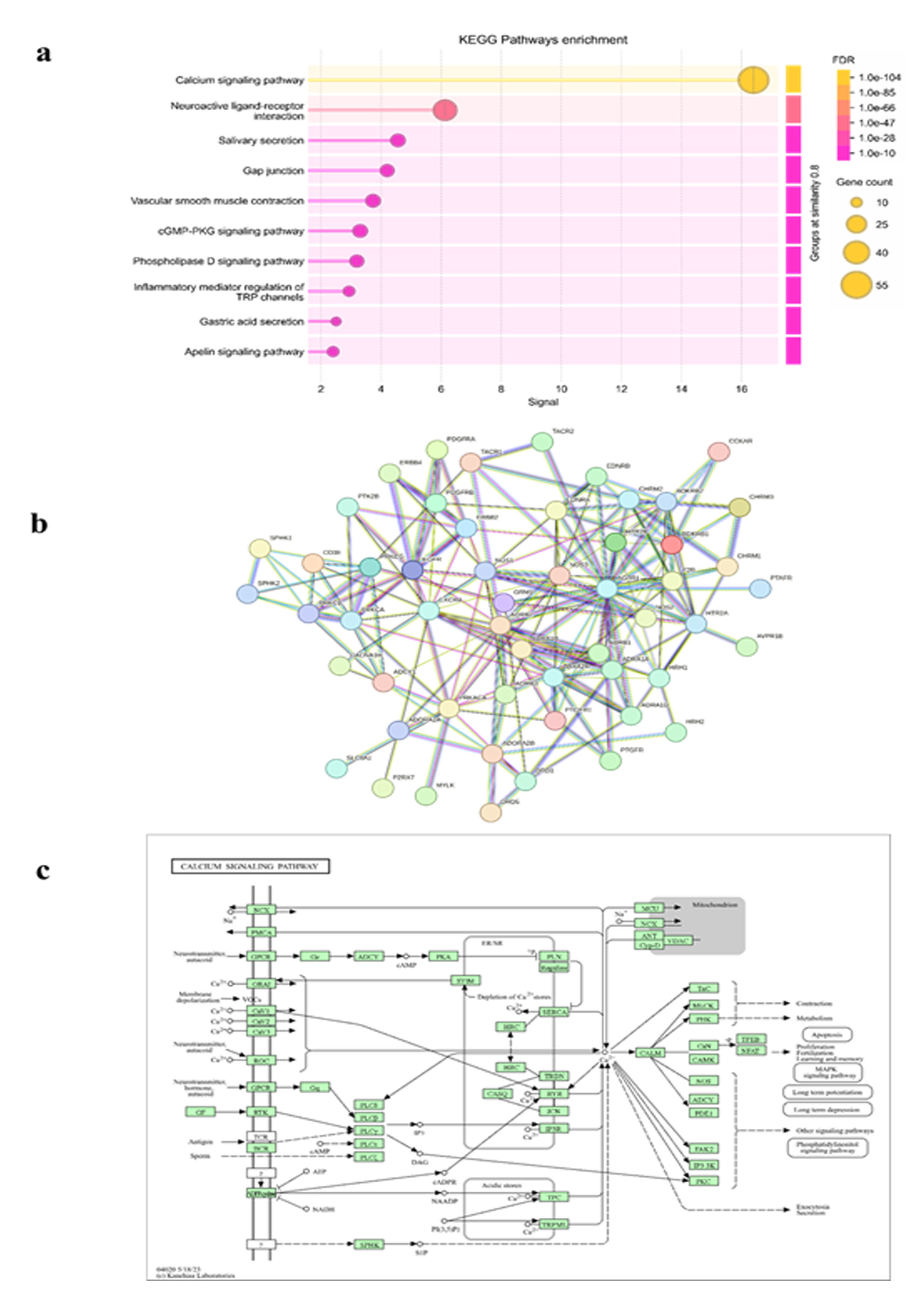

Supplement: S6 Fig — (JPG) [file pone.0346125.s007.jpg]

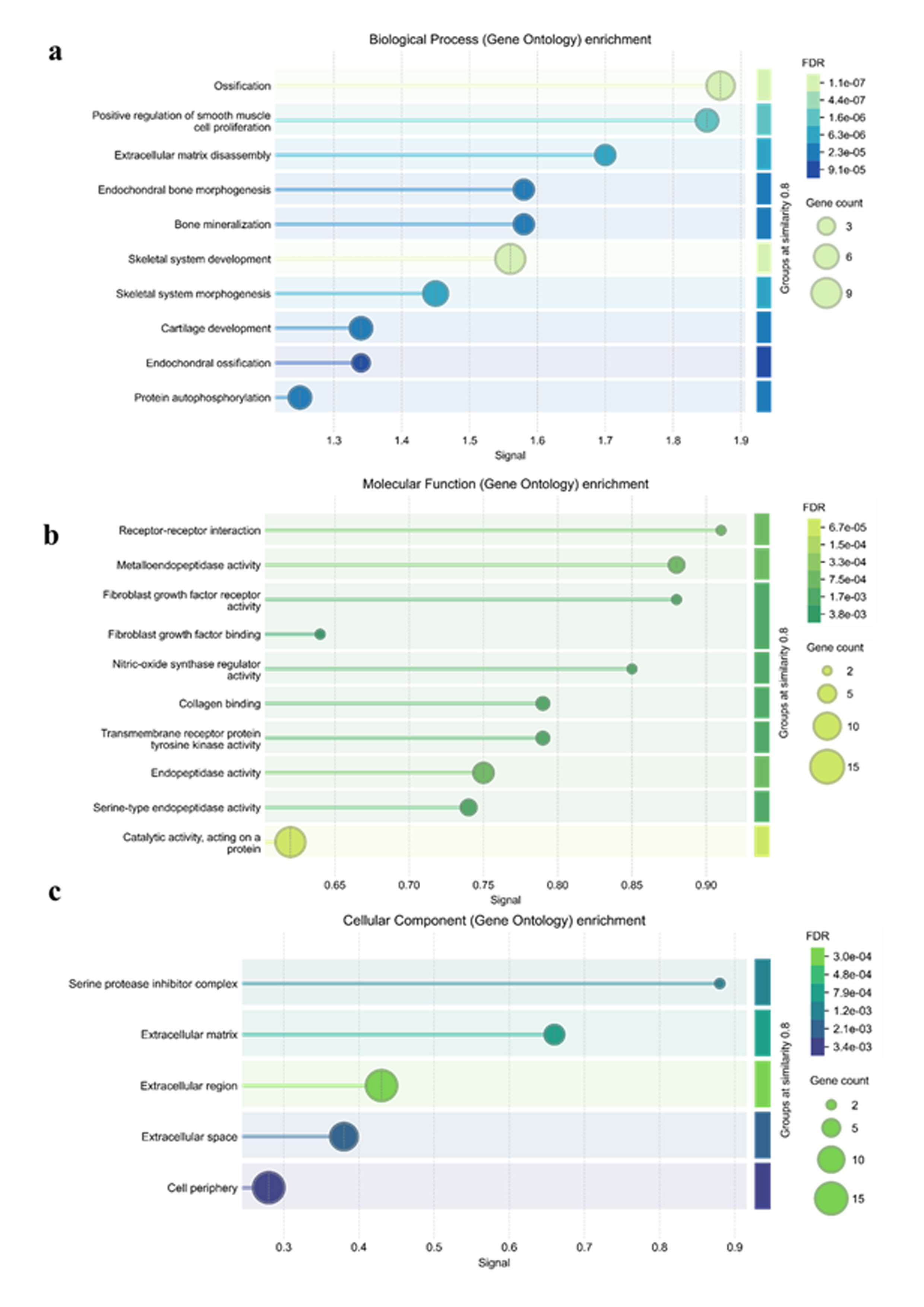

Supplement: S7 Fig — (JPG) [file pone.0346125.s008.jpg]

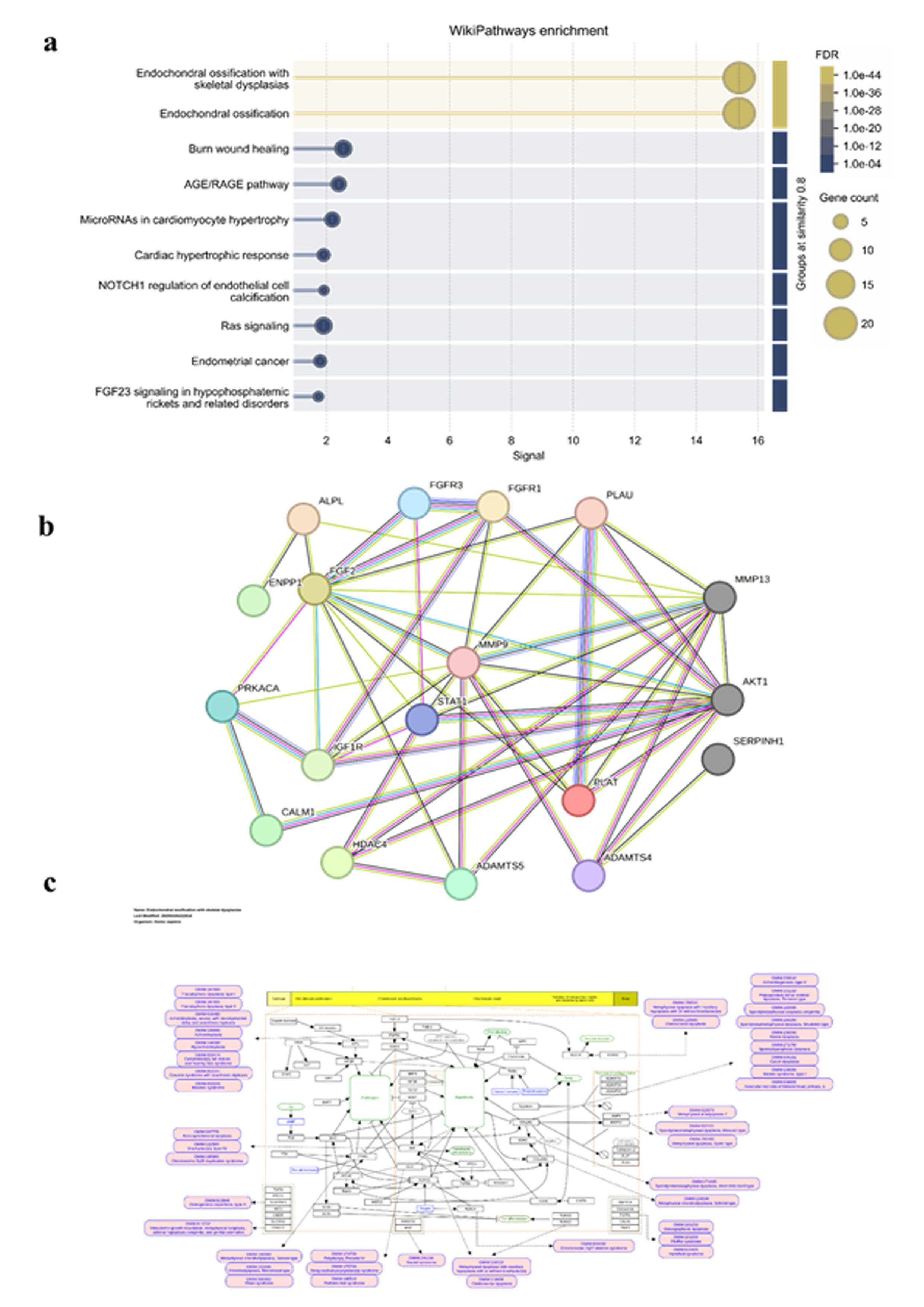

Supplement: S8 Fig — (JPG) [file pone.0346125.s009.jpg]

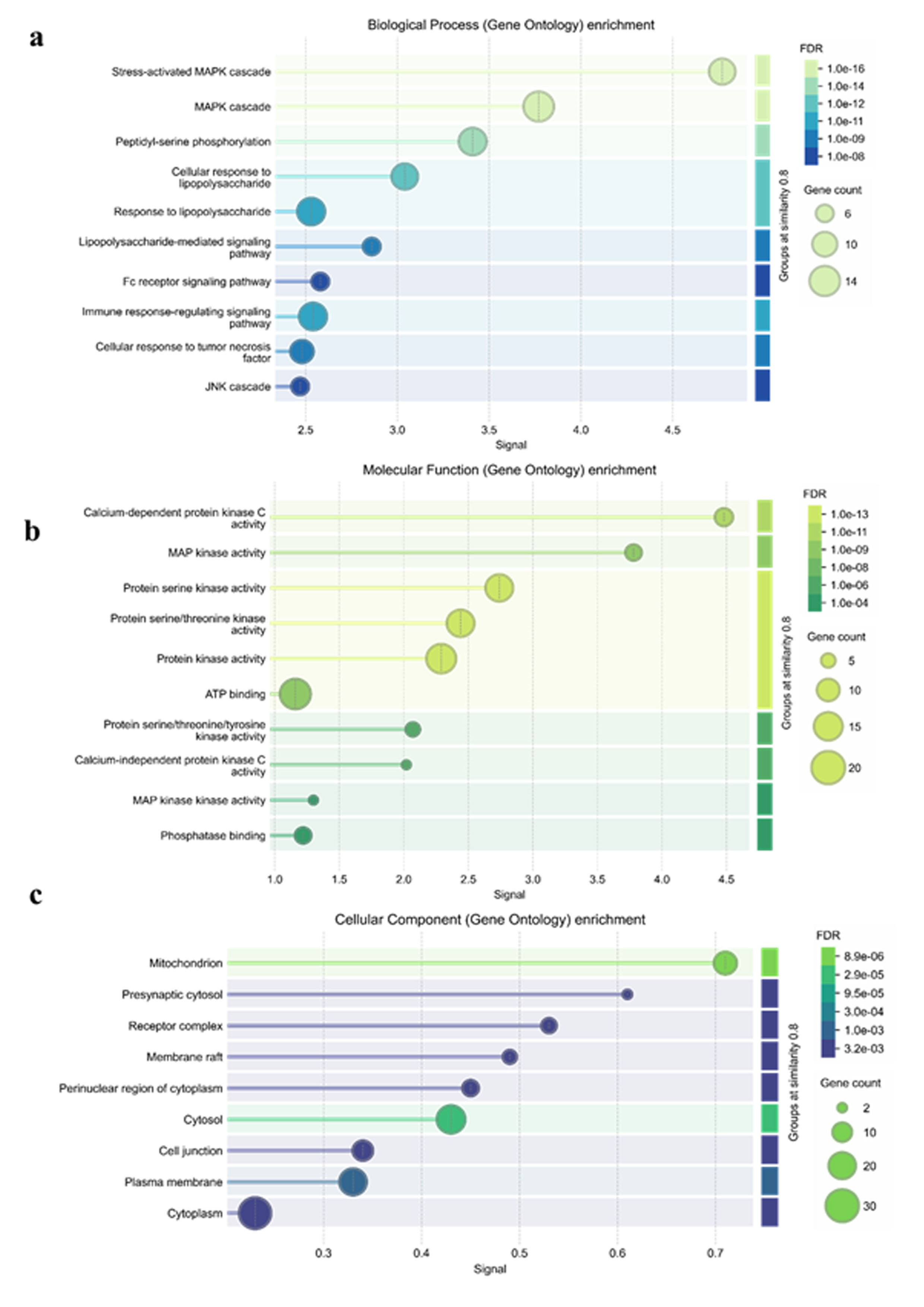

Supplement: S9 Fig — (JPG) [file pone.0346125.s010.jpg]

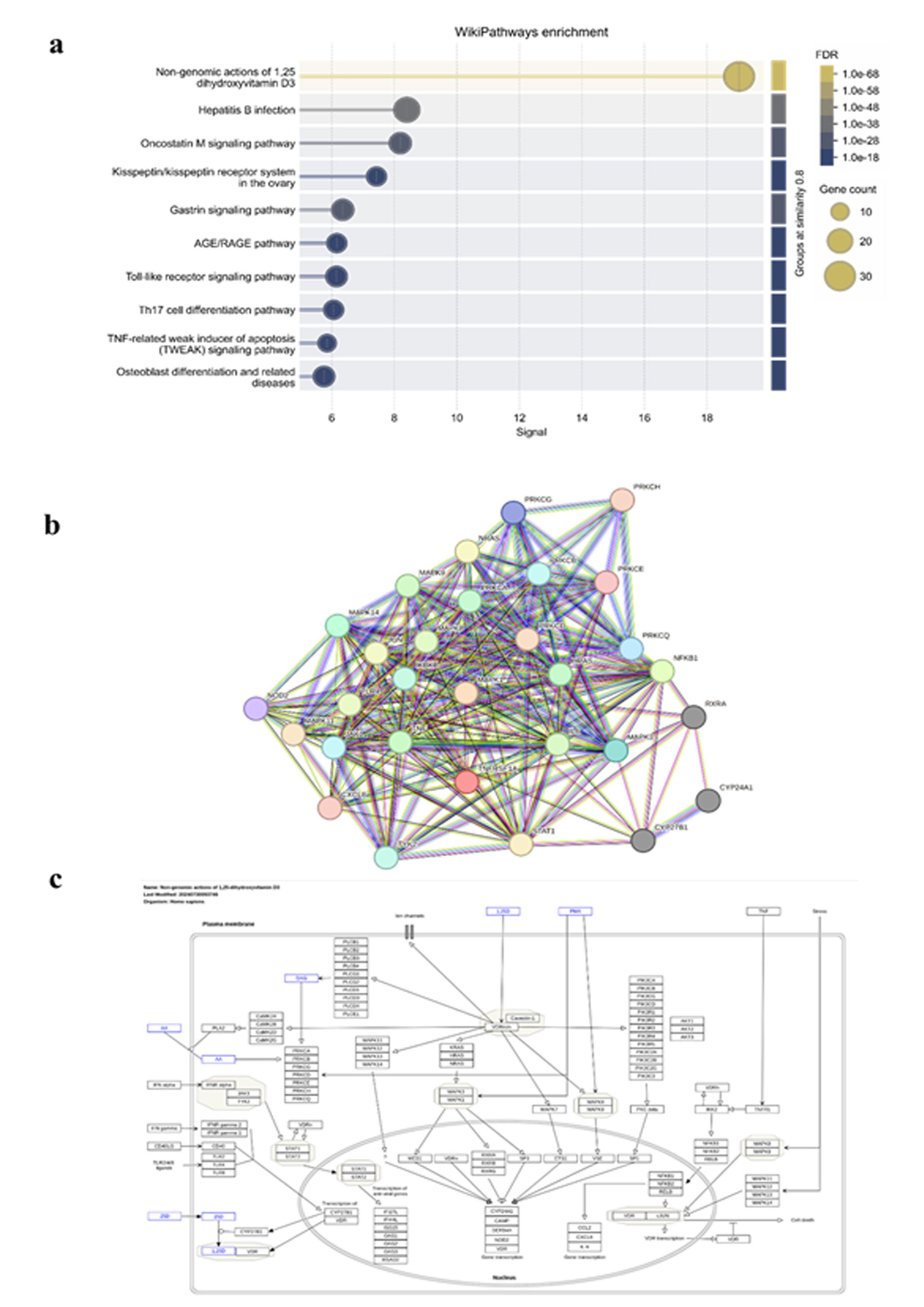

Supplement: S10 Fig — (JPG) [file pone.0346125.s011.jpg]

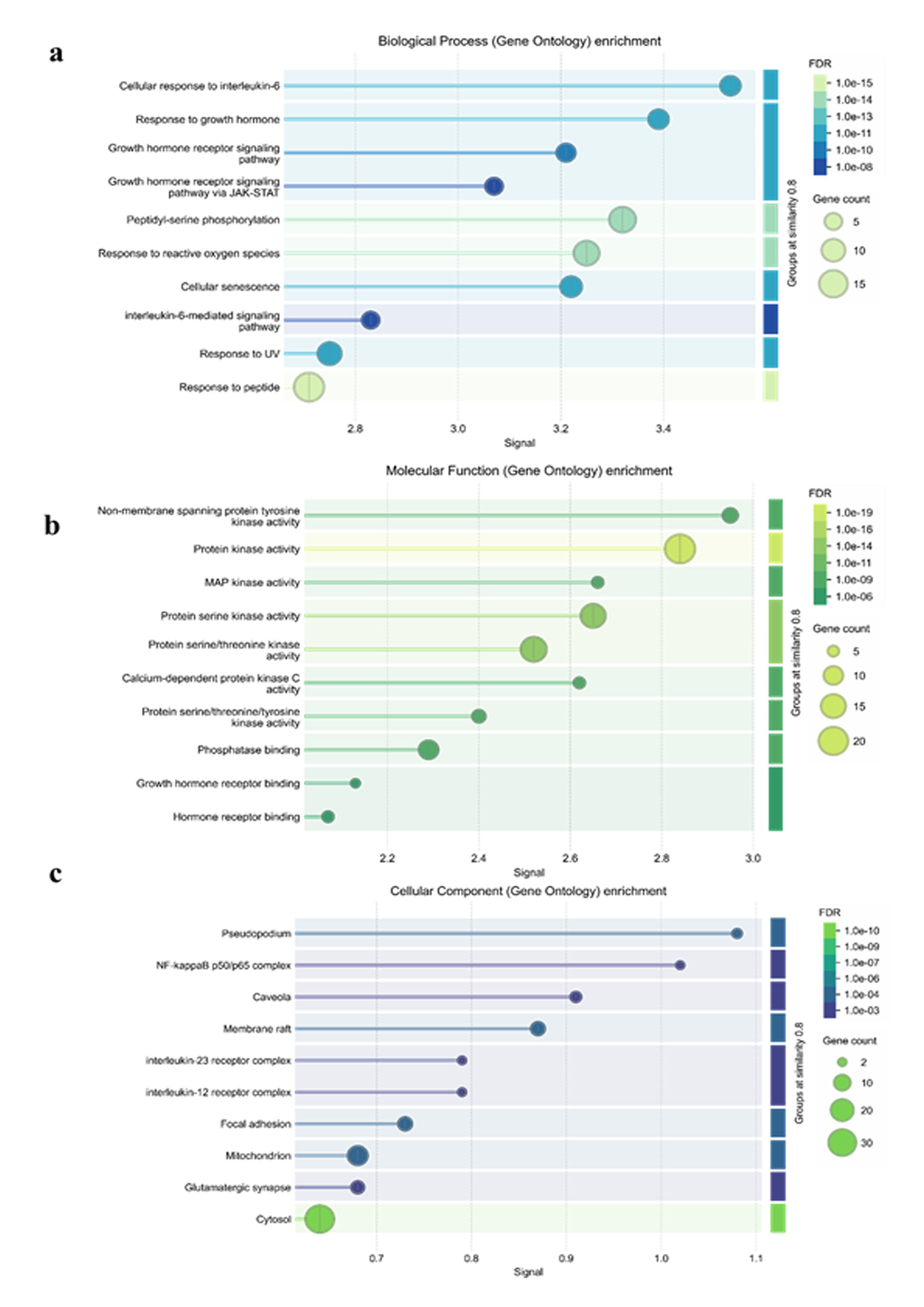

Supplement: S11 Fig — (JPG) [file pone.0346125.s012.jpg]

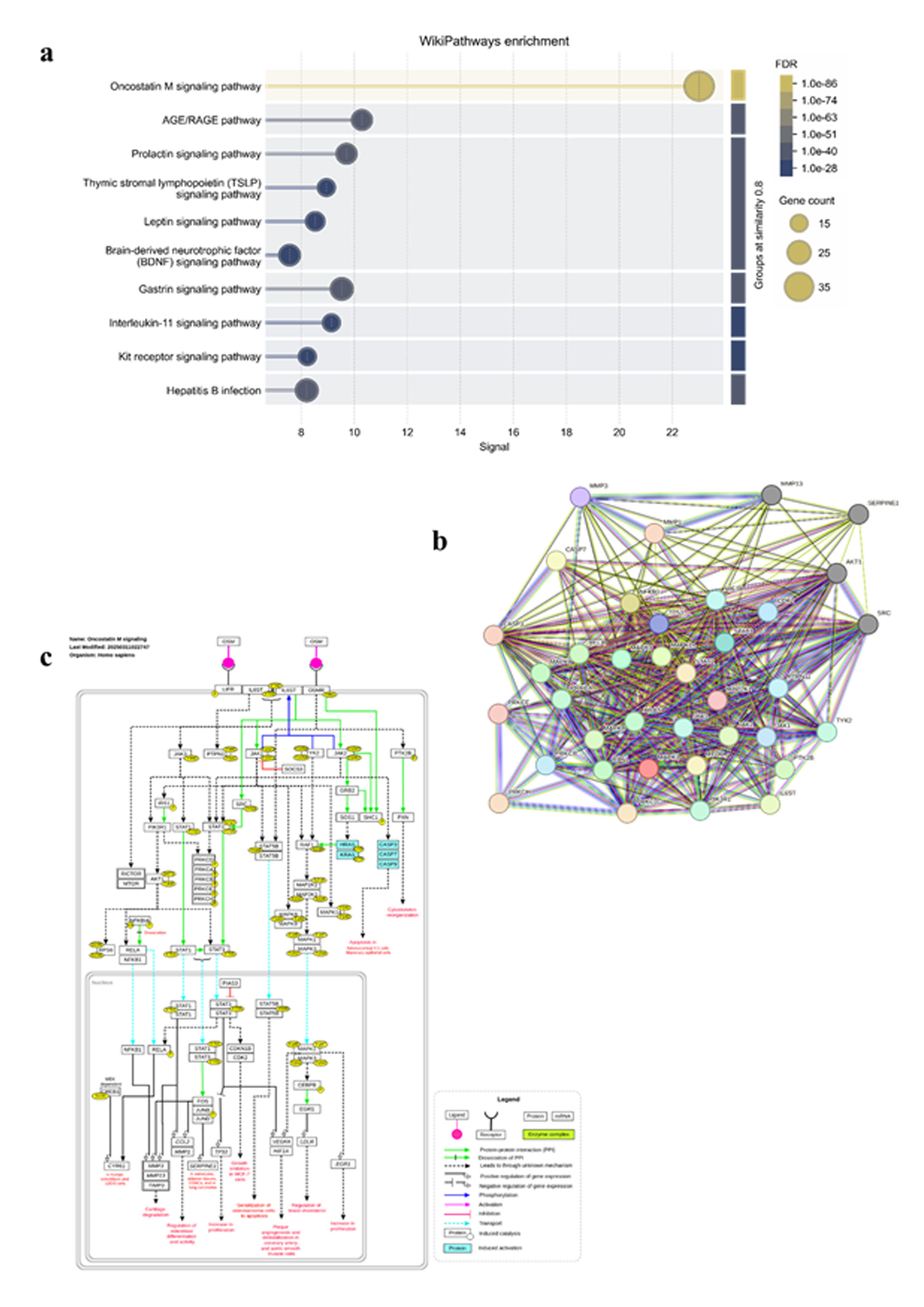

Supplement: S12 Fig — (JPG) [file pone.0346125.s013.jpg]

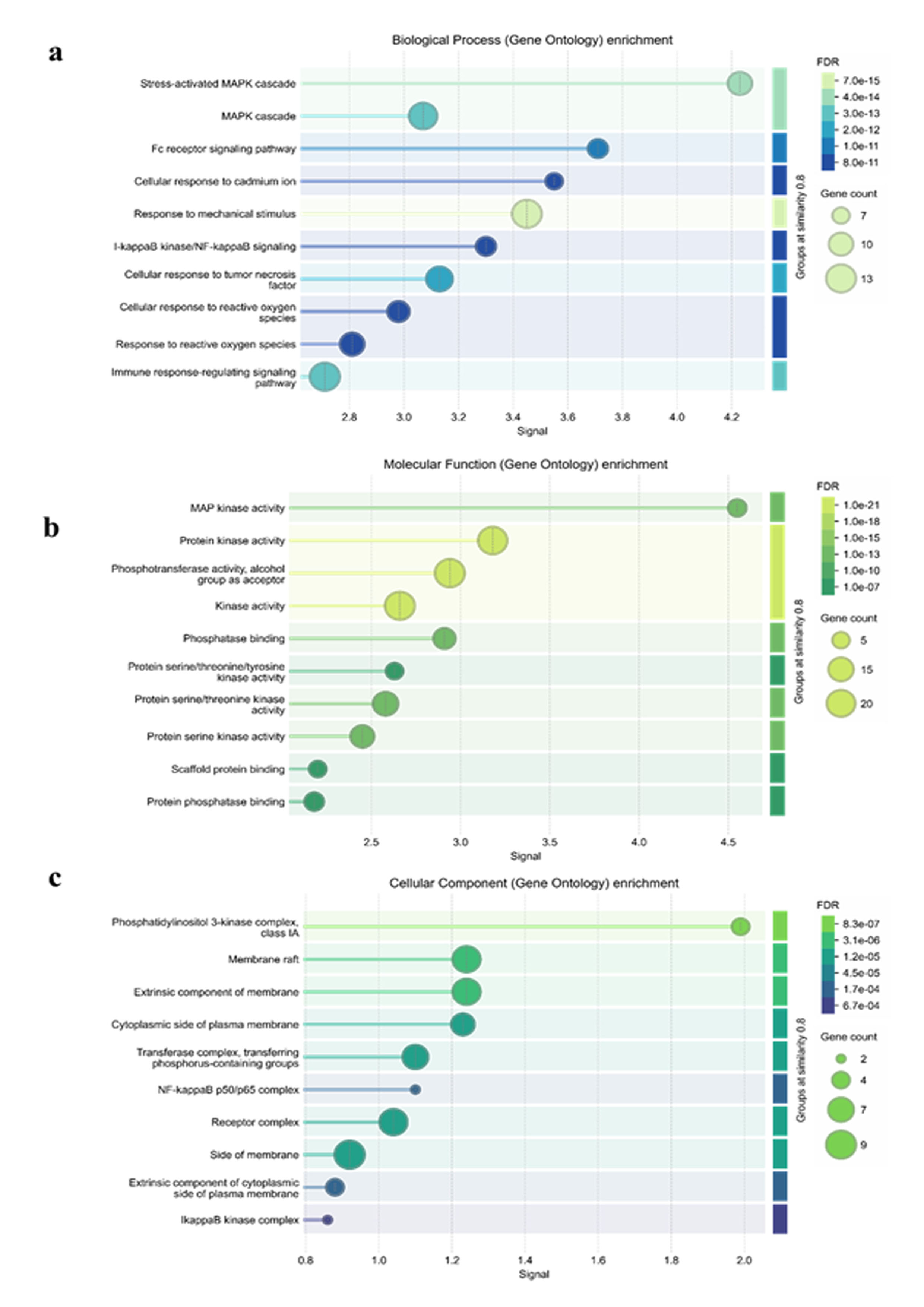

Supplement: S13 Fig — (JPG) [file pone.0346125.s014.jpg]

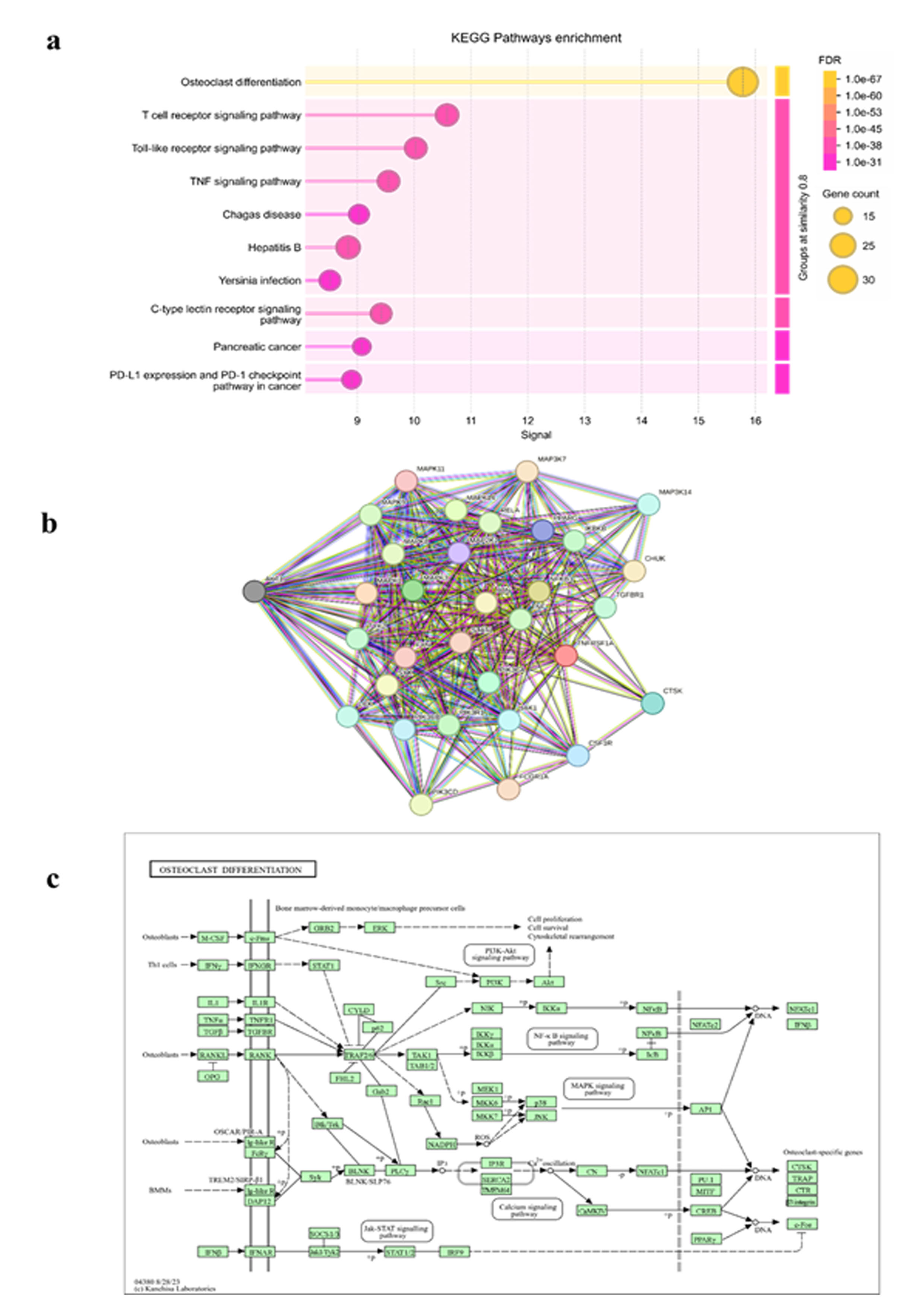

Supplement: S14 Fig — (JPG) [file pone.0346125.s015.jpg]

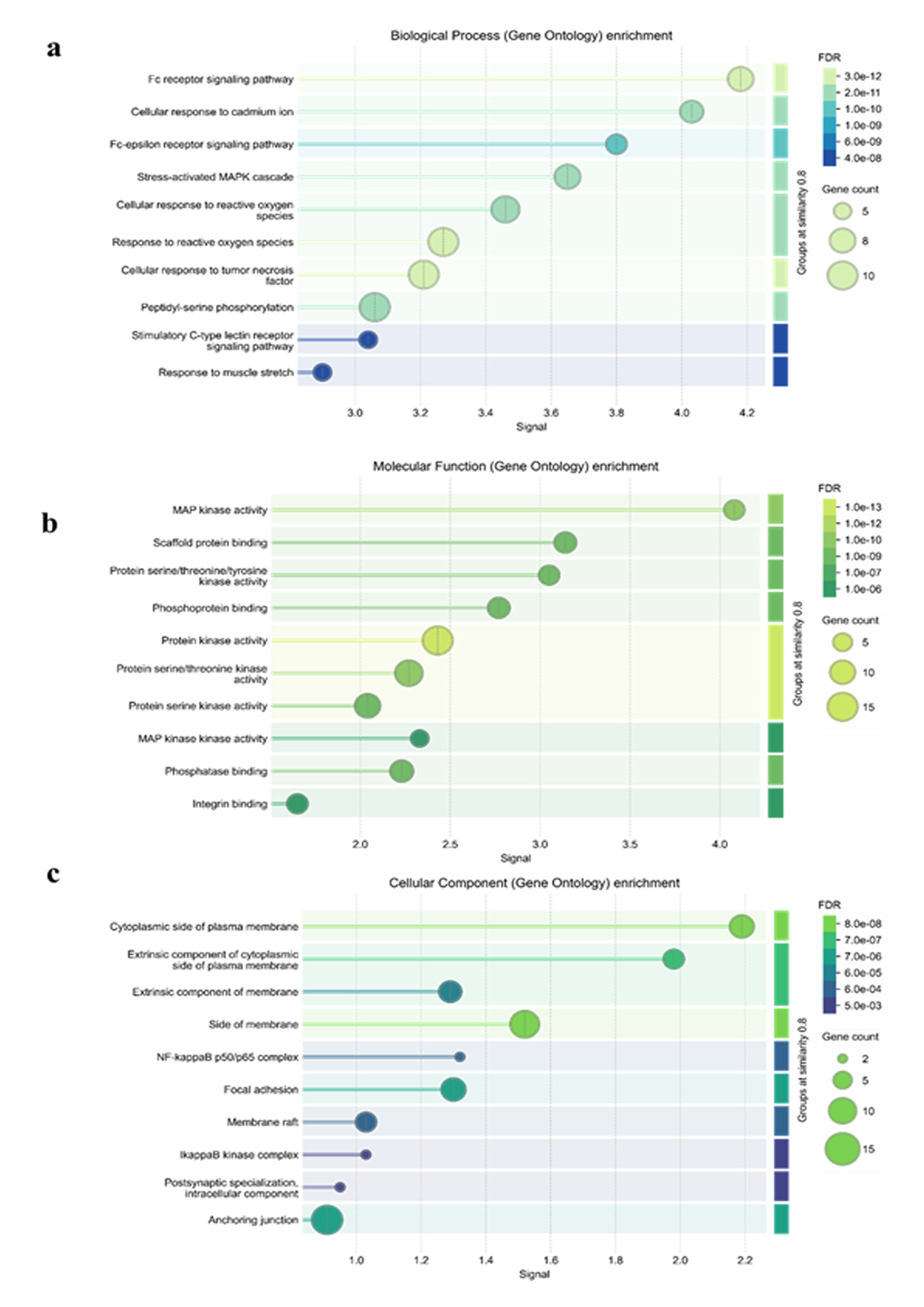

Supplement: S15 Fig — (JPG) [file pone.0346125.s016.jpg]

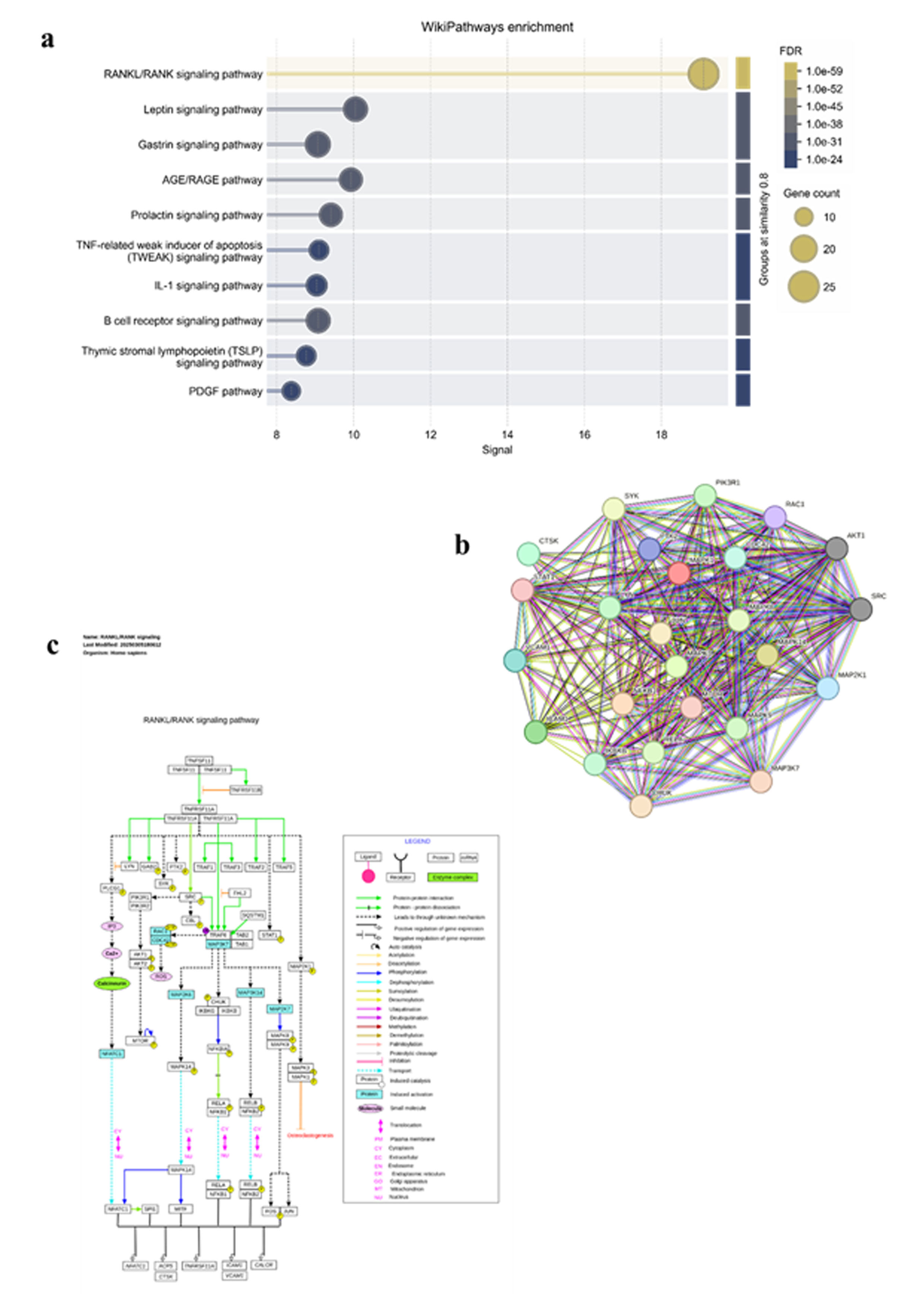

Supplement: S16 Fig — (JPG) [file pone.0346125.s017.jpg]

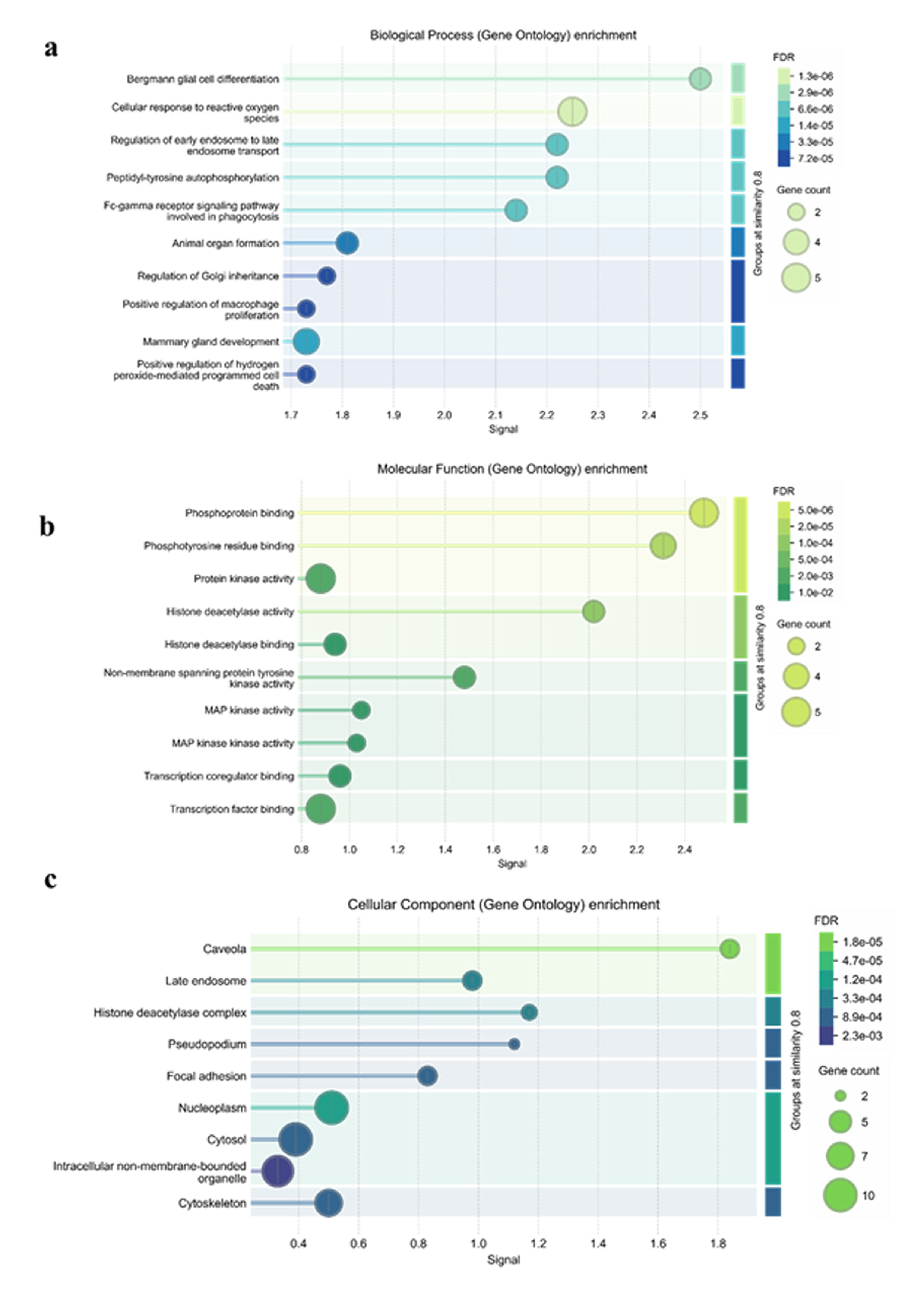

Supplement: S17 Fig — (JPG) [file pone.0346125.s018.jpg]

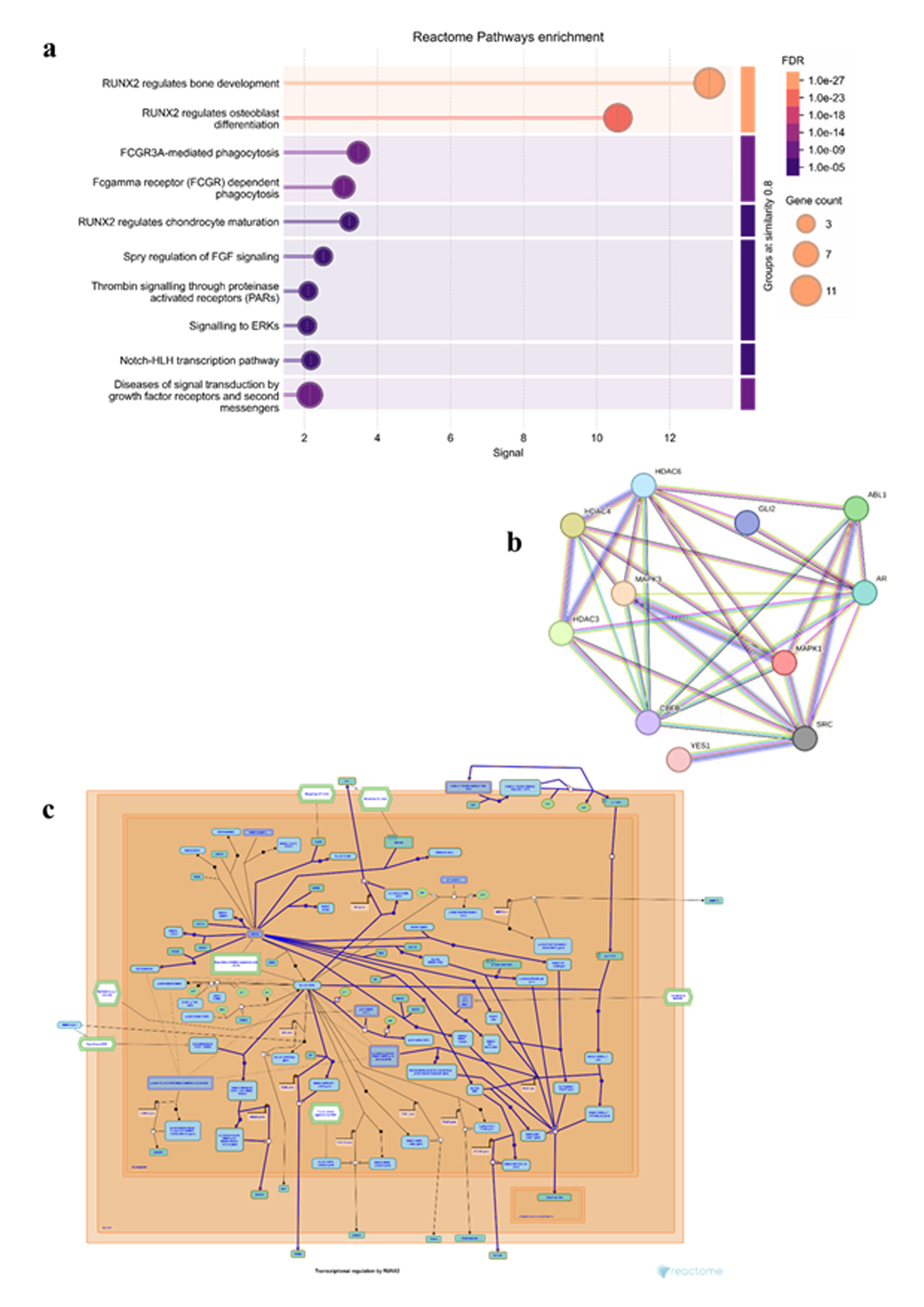

Supplement: S18 Fig — (JPG) [file pone.0346125.s019.jpg]

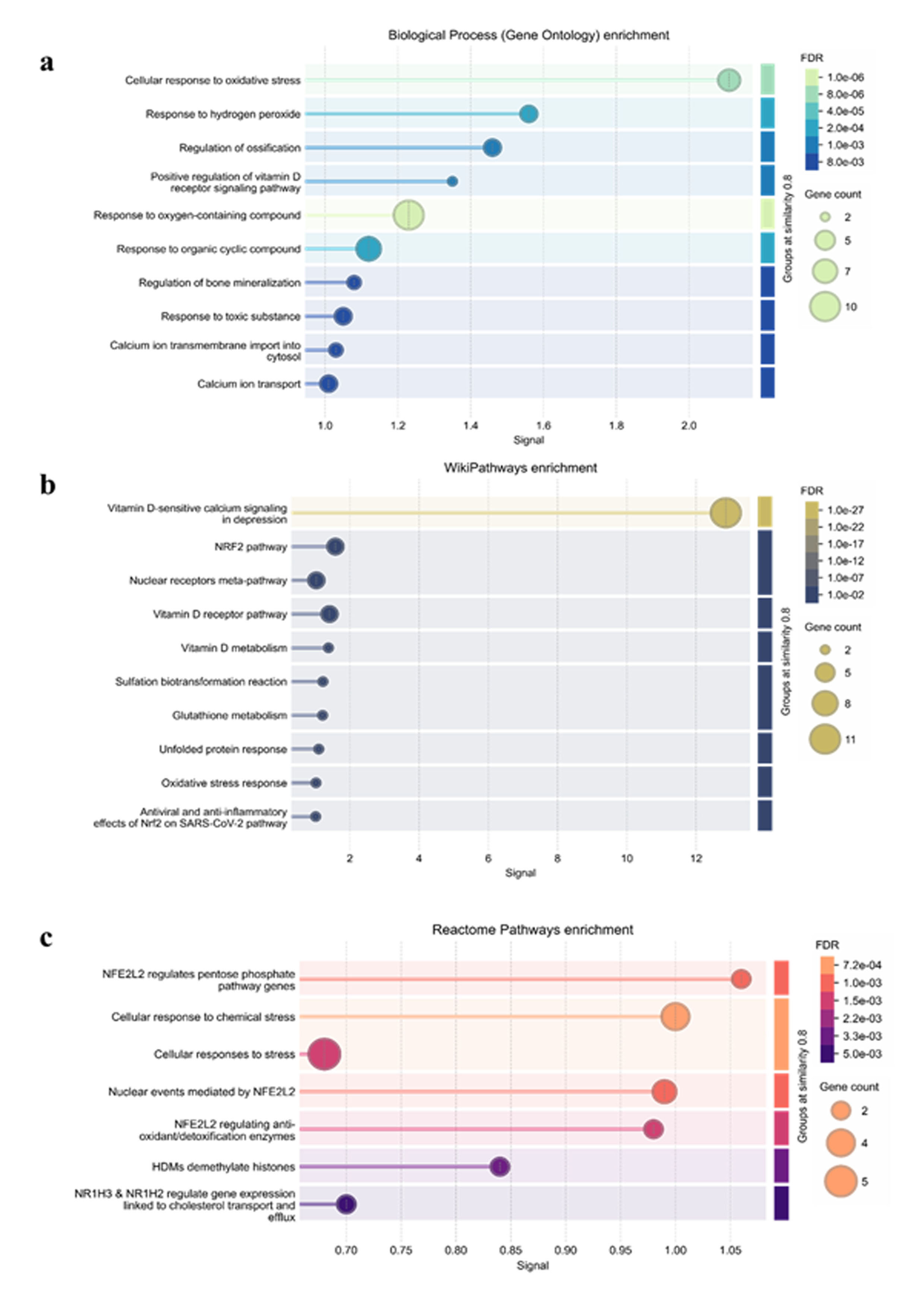

Supplement: S19 Fig — (JPG) [file pone.0346125.s020.jpg]
