# Supplementary material for: Network pharmacological insight into traditional bone healing practices of Sikkim, India
Source: PLoS One. 2026 Apr 15;21(4):e0346125. doi: 10.1371/journal.pone.0346125 (PMC13082723; doi:10.1371/journal.pone.0346125)
Supplement: S1 Table — (DOCX) [file pone.0346125.s021.docx]

**Network Pharmacological Insight into Traditional Bone Healing Practices of Sikkim, India**

Mukunda Anuj Sharma^1,2^, Bharat Gopalrao Somkuwar^3, 22^, Parvin A Barbhuiya^1^, Bhumika Gurung^1^, Madhusmita Mahapatra^1^, Firdous Fatima^3^, Teresa Ningthoujam^1, 22^, Ashika Bhattarai^1^, Bikash Rai^1^, Pravin Kumar^1^, Bishal Tiwari^1^, Sancha Kumar Subba^4^, Zyankit Lepcha^5^, Purna Maya Gurung^6^, Nandalal Khadka^7^, Ratan Bahadur Tamang^8^, Balbir Khati^9^, Shribhakta Chettri^10^, Ran Bahadur Rai^11^, Hem Lall Sharma^12^, Yam Bahadur Rai^13^, Norzang Lepcha^14^, Theptuk Lepcha^15^, Pritiman Singh Chettri^16^, Monraj Limboo^17^, Theweng Gyenthen^18^, Tulshi Pradhan^19^, Prem Gurung^20^, Prem Prashad Dhakal^21^**,** Nanaocha Sharma^1, 22^**,** Lokesh Deb^1, 22*^

1. Biotechnology Research and Innovation Council - Institute of Bioresources and Sustainable Development (BRIC-IBSD) - Regional Centre, Sikkim (Department of Biotechnology, Government of India), 5th Mile, NH-10A, Near Metro Point, Tadong, Gangtok, Sikkim -795001, India.
2. Department of Zoology, Sikkim University, 6^th^ Mile, Tadong, Gangtok, Sikkim, India.
3. Biotechnology Research and Innovation Council-Institute of Bioresources and Sustainable Development - Mizoram (Department of Biotechnology, Government of India), A-1, Nursery Veng, Chawanga Road, Aizawl-796005, India.
4. Traditional Healer, Pachey Village, Samsing, Pakyong, Sikkim – 737106, India.
5. Traditional Healer, Lower Radhu, Dentam, Geyzing, Sikkim, Sikkim - 737113, India.
6. Traditional Healer, Upper Chuba, Phongla, Namchi, Sikkim -737126, India
7. Traditional Healer, Tangzi, Rateypani, Namchi, Sikkim-737126, India
8. Traditional Healer, Dhaje Dhara, Nazi Ruchung, Namchi, Sikkim- 737126, India
9. Traditional Healer, Lower Rangang, Yangyang, Namchi, Sikkim -737134, India
10. Traditional Healer, Namphok, Yangyang, Namchi, Sikkim -737126, India
11. Traditional Healer, Ladam Machong, Pakyong, Sikkim -737131, India
12. Traditional Healer, Assam Daragong, Assam Linzey, Pakyong, Sikkim -737135, India
13. Traditional Healer, Titiribotey, Rorathang, Pakyong, Sikkim 737133, India
14. Traditional Healer, Shipgyer, Mangan, Sikkim -737116, India
15. Traditional Healer, Upper Gor, Lower Dzongu, Mangan, Sikkim -737116, India
16. Traditional Healer, Upper Singhik, Mangan, Near MSSS, Mangan, Sikkim -737116, India
17. Traditional Healer, Mangshila, Upper Ralak, Mangan, Sikkim – 737116, India
18. Traditional Healer, Shakathang, Lachung, Katao Road, Mangan, Sikkim -737120, India
19. Traditional Healer, Lower Timburbong, Soreng, Sikkim – 737121, India
20. Traditional Healer, Namcheybong, Pakyong, Sikkim – 737106, India
21. Traditional Healer, Aritar, Khamdong Singtam, Gangtok, Sikkim-737134, India.
22. Biotechnology Research and Innovation Council - Institute of Bioresources and Sustainable Development (BRIC-IBSD) (Department of Biotechnology, Government of India), Takyelpat, Imphal, Manipur -795001, India.

**Note:** Bharat Gopalrao Somkuwar and Mukunda Anuj Sharma contributed equally

**Short running title:** Traditional Healthcare Practices of Sikkim

***Corresponding address:**

**Lokesh Deb, M. Pharma, Ph.D.**

**Scientist – E (Pharmacology)**

Biotechnology Research and Innovation Council -Institute of Bioresources and Sustainable Development (BRIC-IBSD)– Regional Centre, Sikkim, (Department of Biotechnology, Government of India), 5^th^ mile, NH-10A, Near Metro Point, Tadong, Gangtok -737102,

Sikkim, India; Mob –+919436890969; Email: [lokeshdeb@gmail.com](mailto:lokeshdeb@gmail.com); [lokeshdeb.ibsd@nic.in](mailto:lokeshdeb.ibsd@nic.in)

IBSD Manuscript No. - IBSD/MS/2020/01/12

**Supplementary materials**

**Table S1.** Pharmacology and Phytochemistry of Plants used by Traditional Healers of Sikkim

| Plant no | Scientific name | Common name | Family | Habitat | Part used | Phyto-pharmacology | |
| --- | --- | --- | --- | --- | --- | --- | --- |
|  |  |  |  |  |  | Phytochemicals present | Pharmacological properties |
| 1 | *Viscum articulatum* Burm.f*.* | Harchur | Santalaceae | Parasitic Herbs | Whole plant | lectins, viscotoxins, flavonoids, terpenoids, phenolic acids, and polysaccharides [1] | Anti-inflammatory, anti-cancer, anti-rheumatism arthralgia, anti-cardiovascular diseases, enhancing immunity, and anti-chemotherapy side effects. [1] |
| 2 | *Kaempferia rotunda* L. | Bhui Champa | Zingiberaceae | Herbs | Root | Benzyl benzoate, n-pentadecane, Camphene, Camphor, β-pinene, α-pinene, Linalool oxides, Endo-borneol, Dehydroisoandrosterone acetate, Naphthalene, decahydro-1,1,4a-trimethyl-6-methylene-5-(3- methylene-4-pentenyl) [4aS-(4aα,5β,8aα)], β- phellandrenen, Benzyl benzoate, crotepoxide, 5- hydroxy-7-methoxyflavanone, 7-hydroxy-5-methoxyflavanone7-dihydroxyflavanone, and methyl-D-galactopyranoside [2,3] | Antimicrobial, antielastases, antithyrosinases, antimutagenic, anticancer, antinociceptive, antihyperglycemic, antiallergic, antiandrogenic, anthelmintic, and wound healing [2] |
| 3 | *Astilbe rivularis*Buch.-Ham. ex D.Don | Buro okhati | Saxifragaceae | Herb | Rhizome | Coumarins, aesculetin, astilbic acid, astilbin, aticoside, dimethylaesculetin, daucosterol, eucryphin, palmitine, peltoboykinoleic acid, scopoletin, sitosterol and stilbene, Bergenin and bergenin derivatives, β-amyrin and β-sitosterol, Butanedioic acid, 2,3-bis (8-nonen-1-yl)-, dimethyl ester, Stigmasta-5(6), 22(23)-dien-3-beta-yl acetate [4–6] | Antimicrobial, moderate cytotoxic activity against cancer cell line anti-oxidant, antitumor activity [4,5] |
| 4 | *Bergenia ciliata*(Haw.) Sternb. | Pakhenbet | Saxifragaceae | Herb | Rhizome | bergenin in addition to other phytochemical constituents like afzelechin, catechin, beta-sitosterol, gallic acid, tannic acid, (-)-3-0-galloylepicatechin, (-)-3-0galloylcatechin, gallicin,B. ligulate,β-sitosterol, tannic acid,stigmesterol, gallic acid, bergenin, (+)- afzelechin, (+)-afzelechin, (+)-afzelechintetraccetate, (+)-5,7,4’-trimethoxyafzelechin, (+)-tetramethoxyazelechin, (+)-3-acetyl-5,7,4'-trimethoxyafzelechin [7]. | antibacterial, anti-inflammatory, anticancer, anti-tussive, anti-diabetic, anti-lithotriptic, antimicrobial,Cytoprotective activity, antipyretic activity, Anti-lithiatic activity and nephroprotective effect, Hepatoprotective effect, Anti-malarial effect, Anti-biofilm activity, Anti-arthritis activity [7] |
| 5 | *Fraxinus floribunda* Wall. | Lakuri | Oleaceae | Tree | Bark | 8-acetyl-7-hydroxy-6-methoxycoumarin, 8-methoxycoumarin, 2.5-dihydroxy-6-methoxyacetophenone, fraxetin and aesculetin [8]. | Antioxidant, Anti-inflammatory activity against carrageenan-induced paw edema and Hepatoprotective activity against CCl4-induced hepatotoxicity in Wistar rats and Antidiabetic Anti-nociceptive activity, anti-arthritic activity [9–11]. |
| 6 | *Euphorbia hirta*L. | Bhui Chipley | Euphorbiaceae | Herb | Root | rutin, quercitin, euphorbin-A, euphorbin-B, euphorbin-C, euphorbin-D, chlorophenolic acid, 2,4,6-tri-O-galloyl-β-d-glucose, 1,3,4,6-tetra-O-galloyl-β-d-glucose, kaempferol, gallic acid, and protocatechuic acid, β-amyrin, 24-methylenecycloartenol, β-sitosterol, heptacosane, nonacosane hikmic acid, tinyatoxin, choline, camphol, and quercitol derivatives containing rhamnose and chtolphenolic acid, chlorophenolic acid, leucocyanidin, myricitrin, cyaniding 3,5diglucoside, camphol, flavonol, inositol, tetraxerol, β-sitosterol, and kaempferol, Afzelin, euphorbin-A, euphorbin-B, euphorbin-C, euphorbin-D, gallic acid, and protocatechuic acid [12–14] | Antibacterial, antimalarial, anti-inflammatory, galactogenic, Antiasthmatic, Antidiarrheal, Antioxidant, Antifertility, Antiamoebic, Antifungal activity [13]. |
| 7 | *Prunus cerasoides*Buch.-Ham. ex D.Don | Payum | Rosaceae | Tree | Bark | dihydrotectochrysin, pinocembrin, dihydrowogonin, chrysin, naringenin, kaempferol47, aromadendrin, quercetin, taxifolin, Carasinone, Carasidin, Carasin, narigenin, apigenin, β-sitosterol, sakuranetin, prunetin, genkwanin, β-sitosterol, ursolic acid, oleic, palmitic, stearic acids, afzelin, kaempteritrin, naringenin, β-sitosterol-β-D-glucoside, Puddumin-B, Padmakastein, β-sitosterol behenate, tectochrysin, genistein, leucocynidin, 4´-glucoside of genkwanin, chrysophenol, Quercetin-3-rhamnoglucoside, kaempferol [15]. | anti-melanogenic, anti-plasmodial activity, Fruit extract shows anti-inflammatory activity by modulating iNOS pathway and Th1/Th2 immune homeostasis in activated murine macrophages and lymphocytes [16–18]. |
| 8 | *Macropanax dispermus* (Blume) Kuntze | Pachpatey | Araliaceae | shrub | Bark | *Macropanax dispermus* (Wallich ex G. Don) Seemanncontains vitamin E& C, carotene, xanthophylls, tannins, phenolicsproperties [19]. | *Macropanax dispermus* (Wallich ex G. Don) Seemann crude extract shows thrombolytic, cytotoxic, analgesic, and antipyretic properties [19]. |
| 9 | *Urtica parviflora*Roxb. | Gharia sisnu | Urticaceae | Herb | Root |  | Alpha amylase & alpha-glucosidase inhibitory activity [20]. |
| 10 | *Saurauia napaulensis*DC. | Gagun | Actinidiaceae | Shrub | Bark |  | Treatment of fever and viral disease [21]. |
| 11 | *Rubus ellipticus* Sm. | Aishleo | Rosaceae | Shrub | Whole plant |  |  |
| 12 | *Mimosa rubicaulis*Lam. | Ararey | Fabaceae | Shrub | Root | Quercetin, luteolin, 5,7,4 '-trihydroxy-6,3',5'trimethoxy-flavone,7-O-alpha-Larabinopyranosyl(1->6)-O-beta-Dlucopyranoside, 4ethylgallic acid, Triterpenes, steroids, flavonoids, tannins, carbohydrates and alkaloids Β-Sitosterol, 4-Dihydoxy-3-methoxy cinnamic acid, Octadecanoic acid [22]. | anti-inflammatory, anti-oxidant and analgesic activity also used in the treatment of burns, roots as an antiemetic agent. [23,24] |
| 13 | *Artocarpus lakoocha*Roxb. | Barar | Moraceae | Tree | Bark | Artocarpin (α -D-galactosyl-binding lectin), Oxyresveratrol [25] | Anti-inflammatory, antibacterial, antioxidant, antiviral, antiproliferative activity also inhibit α-glucosidase and neuraminidase, and cytotoxic activities against different cancer cell lines [26,27]. |
| 14 | *Girardinia diversifolia*(Link) Friis | Bhagresisnu | Urticaceae | Shrub | Root | β-sitosterol, 7-hydroxysitosterol and 3-hydroxystigmast-5-en-7-one (Njogu et al.,2011), Quinic acid, Citric acid, Gluconic acid, 3-O-Caffeoyl quinic acid, Synapoilquinic acid,vitexin, Ganoderic acid, Phytol, Stearic acid, Lignoceric acid, Cerotic acid, Uvaol, γ-Sitosterol, α-Tocopherylquinone, β-sitosterol, α-Cryptoxanthin, Carotenoid, Phytoene Scopoletin [28]. | Antimicrobial, inhibittyrosinase, α-amylase and α-glucosidase, acetylcholinesterase (AChE), butyrilcholinesterase (BuChE) and also used in asthma, gastritis, headache, joint pain and tuberculosis [28,29]. |
| 15 | *Trichosanthes cucumerina*L*.* | Bhaishe shig | Cucurbitaceae | Herb | Root | Vitamin – A, C & E, phenols, flavonoids.  Cucurbitacin-B & E, β-sitosterol, Stigmasterol etc. [30,31] | Antioxidant, anticancer, anti-inflammatory, anti-dandruff, anti-fertility, antihepatotoxic, antidiabetic activity, Antifungal, Antibacterial and gastroprotective [30,31] |
| 16 | *Clinopodium umbrosum*(M.Bieb.) K.Koch | BillaJhora | Lamiaceae | Herb | Whole plant | buddlejasaponin IV and buddlejasaponin IV [32] | Shows anticancer/cytotoxicity against HN-5 cells [32] |
| 17 | *Ficus benghalensis*L. | Bor (patty) Seti bar | Moraceae | Tree | Bark | quinic acid, Palmitic acid, methyl ester, Eicosadienoic acid, ergosterol acetate and α-amyrenyl acetate, Lupenyl acetate and α-amyrenyl acetate, Globulol, Phytol, Stearic acid, Sitosterol, Lupeol, Amyrin acetate, Lupenyl acetate, Friedelanol, Cyclolaudenol, 1-Heptatriacotanol, Furostanol Stigmasterol,Nonanoic acid, Quinic acid, Neophytadiene, Octadecatrienoic acid, Hexadecanoic acid [33,34] | Antioxidant, anti-inflammatory, anticancer activity, [34]. |
| 18 | *Lepidium sativum*L. | Chausor | Brassicaceae | Herb | Whole Plant & Seed | Glucosinolates, benzyl isothiocyanate, α-pinene, and phenyl acetonitrile, benzyl isothiocyanate, α-pinene, palmitic acid, linoleic acid, benzyl isothiocyanate, α-pinene, palmitic acid, phenyl acetonitrile, sabinene, and limonene, β-thujone, campesterol, cis-vaccenic acid, 2-naphthalenol, Sinapine, Ascorbic acid, Apigenin, Luteolin, (E)-p-coumaric acid, Kaempferol, Chlorogenic acid, 6-prenylnaringenin, Hexose rhamnose 1 [35]. | Possess antimicrobial, anticancer, antioxidant, anti-inflammatory and fracture/ bone healing activity also used as bronchodilator, hypotensive, hypoglycemic, hepatoprotective agents [35,36]. |
| 19 | *Heracleum wallichii*DC. | Chimping | Apiaceae | Herb | Flower | Bisbenzylisoquinoline alkaloids, cycleanine, isochondrodendrine, stigmasterol dihydrofuranocoumarinns, columbianetin, marmesin and vaginidiol. [37]. | Anti-inflammatory, antimicrobial, anticholinesterase, antioxidant, antiviral, cytotoxic anticarcinogenic, antidiarrheal activity [38] |
| 20 | *Schima wallichii*(DC.) Korth. | Chowleney | Theaceae | Tree | Bark |  | Antimicrobial, anticoagulant, and antioxidant properties and shows anticancer activity against HeLa cells, and also possess anti-inflammatory response in carrageenan-induced paw oedema and cotton pallet granuloma, respectively [39–41] |
| 21 | *Zingiber montanum*(J.Koenig) Link ex A.Dietr. | Fakchem | Zingiberaceae | Herbs | Rhizome | α-Zingiberene, β-Bisabolene, α-Pinene, Sabinene, β-Myrcene α-Terpinene, p-Cymene, β-Ocimene, γ-Terpinene, 4-Thujanol, α-Terpinolene, trans-4-Thujanol, cis-p-Menth-2-en-1-ol, trans-p-Menth-2-en-1-ol, Terpinene-4-ol, γ-Terpineol, γ-Terpineol acetate, 2-Camphanol acetate, p-cymene-7-ol, Terpinene 4-acetate, β-Elemen, Isovanillin, γ-Elemene, β-Sesquiphellandrene, Asarone, iphenylbutenoids [42] | Anti-inflammatory and acetylcholinesterase inhibitory activity and also shows anti-hypercholesterolemic effects in HFD-treated Wistar rats [43,44] |
| 22 | *Centella asiatica*(L.) Urb. | Golpatta | Apiaceae | Herb | Whole plant | Madecassoside, Asiaticoside, Madecassicacid, Madecassicacid Valine, Triparanol, butamben, ivermectin, neuraminic acid, aesculin, esculetin, famciclovir, isocitretin, rhoifoline, gentiopicrin, pelargonic acid, urocortisone gabapentin, sarmentoside, khivorin,Asiaticoside, madecassic acid, madecassoside, asiatic acid, Asiaticoside, Asiatic acid, asiaticoside [45]. | Effective against neurological disorder, Rheumatoid arthritis, Type2 diabetes mellitus, Diabetic neuropathy, Hyperlipidemic, Osteoporosis, Acne, Incision and burn, Liverfibrosis, Coloncancer, Gastriculcers, Pelvic inflammation, Submucous fibrosis [46,47] |
| 23 | *Ficus virens*Aiton. | Kabra (Ficus) | Moraceae | Tree | Bark | 2-Hydroxycyclopent-2-En-1-One, ElaidoicAcid, n-Hexadecanoicacid, 1-Docosene, quinicacid, anozo, caryophyllene, eugenol, phosphonic acid, catechol, butyl carbitol, alletone, ϒ-caryophyllene, Pravastatin [48]. | Antityrosinase, Antioxidative and β-hydroxy β-methylglutaryl-CoA Reductase Inhibitory activity [48,49] |
| 24 | *Curcuma caesia*Roxb*.* | Kalo haldi | Zingiberaceae | Herb | Rhizome | 2-Nonanol, Camphor, Isobomeol, Endo-Borneol, γ-Elemene, β-Copaene, Curzerene, Coniferol, Himbaccol, Boldione, Spathulenol, α-Eudesmol, α-Santonin, Ivalin, Confertin, Cadinene, Rotundene, Xanthinin, Velleral, Acorenol, Velleral, Arglabin [50]. | Antioxidant, antimicrobial, antiproliferative, anticancer, anti-inflammatory, antiulcer, antidiabetic, Neuropharmacological properties [51] |
| 25 | *Clerodendrum infortunatum*L. | kalochito | Lamiaceae | Shrub | Whole plant | β-sitosterol, stigmasterol, retusin, oleanolicacid, ayanin, quercetin [52] | Shows anticancer activity against HPV-positive cervical cancer and also shows highest cytotoxicity against tumor cell line and possess dose-dependent anti-inflammatory activity against e carrageenan, histamine and dextran-induced rat paw edema [52] |
| 26 | *Abelmoschus manihot*(L.) Medik. | kapasay/jangli vindi | Malvaceae | Shrub | Root | Myricetin, Quercetin, Isoquercitrin, Scopoletin, Glycerolmonopalmitate, protocatechuicacid, caffeicacid, palmiticacid, hexacosoicacid, gallicacid. Volatiles include maleic acid, tetracosane, hexadecane, heneicosane, octadecane, allyl undecylenate, docosane, hexadecanoic acid, tetradecanoic acid, undecanone [53] | Antidiabetic, antioxidant, anti-inflammatory and analgesic activity also used as antidepressant, neuroprotective, antiviral, antitumor, immunomodulatory, cardioprotective, proangiogenic agents [53] |
| 27 | *Euodia meliifolia*(Hance) Benth. | Khanakpa | Rutaceae | Tree | Bark | evodiamide [54] |  |
| 28 | *Engelhardia spicata*Lechen ex Blume | Mauwa | Juglandaceae | Tree | Bark | Spicata, Eucryphin, Quercetin-3-O-[β-D-glucopyranosyl (1→4)-β-D-xylopyranosyl (1→4)]-α-L-rhamnopyranoside, 3,5,7-Trihydroxychromone [55] | Ethnomedicine used in Asia for treatment of fever, detoxication, rheumatism, diarrhoea and  shows antidiabetic activity in alloxan-induced diabetic mouse [55] |
| 29 | *Santalum album*L*.* | Molagiri | Santalaceae | Tree | Bark | α-santalol, β-santalol, santene, nortricyclo-ekasantalene, teresantalol, nor-tricyclo-kasantalal, α-and β- santalic acids, ketosantali acid, betulinic acid, β-sitosterol [32] | Anti-fungal, anti-bacterial, anti-cancer, antioxidant, anti-ulcerogenic, and also possess anti-inflammatory activity against formalin-induced paw edema and yeast-induced pyrexia in albino rats [32,56] |
| 30 | *Rheum nobile*Hook.f. & Thomson | Padamchal | Polygonaceae | Herb | Rhizome | Anthraquinones, Rheumone A, stilbene glycoside, piceatannol-4′-*O*-*β*-D-glucosid, resveratrol-4′-*O*-*β*-D-glucoside, (-)-epiafzelechin, (-)-epicatechin-3-*O*-gallate, emodin-8-*O*-*β*-D- glucopyranoside, (+)-catechin torachrysone-8-*O*-*β*-D-glucopyranoside, (-)-epicatechin, emodin, chrysophanol, chrysophanol-8-*O*-*β*-D-glucopyranoside, physcion-8-*O*-*β*-D-glucopyranoside, *trans*- emodindianthrone and C*is*-emodindianthrone [57] | Shows antioxidant and anti-inflammatory activity by inhibiting HRBC haemolysis and protein denaturation [57,58] |
| 31 | *Ficus elastica*Roxb. ex Hornem. | Labar | Moraceae | Tree | Bark | Oleanolic acid, Ursolic acid, Friedelin, Friedelinol, Betulinic acid, Sitosterol 3-O-ß-D- glucopyranoside, Morin, Quercitrin, Myricitrin, Biochanin A, Feroxidin, Ficus elastic acid, Chlorogenic acid, Emodin, Elastiquinone, Rutin, Kaempferin, Syringin, Elasticoside, Ficusoside, Ficusamide. [59] | Antioxidant, anticancer, anti-inflammatory and Anti-proliferative activity [59,60] |
| 32 | *Betula alnoides*Buch.-Ham. ex D.Don | Saur | Betulaceae | Tree | Bark | betulinic acid, botulin, lupeol, oleanolic acid, ursolic acid [61](Chaniad et al., 2019) | Antioxidant, antimicrobial and antidiabetic activity and also shows anti-austerity activities against human pancreatic cancer cell line PANC‑1 [62,63]. |

**References:**

1. Song C, Wei X-Y, Qiu Z-D, Gong L, Chen Z-Y, Ma Y, et al. Exploring the resources of the genus *Viscum* for potential therapeutic applications. Journal of Ethnopharmacology. 2021;277: 114233. doi:10.1016/j.jep.2021.114233

2. Aryantini D, Astuti P, Yuniarti N, Wahyuono S. Extraction and Isolation of Phytochemicals from Kaempferia rotunda Linn. (White Turmeric) for Pharmacological Application: A Review. Tropical Journal of Natural Product Research. 2022;6(9): 1359–1366.

3. Sahoo S, Lenka J, Kar B, Nayak S. Clonal fidelity and phytochemical analysis of in vitro propagated Kaempferia rotunda Linn.—an endangered medicinal plant. In Vitro CellDevBiol-Plant. 2023;59: 329–339. doi:10.1007/s11627-023-10342-8

4. Rai V, Pogu SV, Bhatnagar R, Bomzan P, Dutta A, Mandal A, et al. Biological evaluation of a natural steroid ester, Stigmasta-5(6), 22(23)-dien-3-beta-yl acetate isolated from the Himalayan herb *Astilbe rivularis* as potential antitumor agent. Chemico-Biological Interactions. 2022;360: 109935. doi:10.1016/j.cbi.2022.109935

5. Rai V, Kumar A, Das V, Ghosh S. Evaluation of chemical constituents and in vitro antimicrobial, antioxidant and cytotoxicity potential of rhizome of Astilbe rivularis (Bodho-okhati), an indigenous medicinal plant from Eastern Himalayan region of India. BMC Complement Altern Med. 2019;19: 200. doi:10.1186/s12906-019-2621-6

6. Timalsena S, Lamichhane PP. Astible Rivularis: Bioactive Compounds and Pharmacological Functions. Chin J Integr Med. 2019;25: 795–799. doi:10.1007/s11655-016-2260-4

7. Kour H, Raina R, Verma PK, Khan AM, Bhat MA, Nashiruddullah N. Evaluation of the wound healing activity of ethanolic extract of *Bergenia ciliata* (Haw.) Sternb. rhizome with excision wound model in Wistar rats. Journal of Ethnopharmacology. 2021;281: 114527. doi:10.1016/j.jep.2021.114527

8. Nagarajan GR, Rani U, Parmar VS. Coumarins from Fraxinus floribunda leaves. Phytochemistry. 1980;19: 2494–2495. doi:10.1016/S0031-9422(00)91068-9

9. Lingadurai S, Nath LK, Kar PK, Besra SE, Joseph RV. Anti-Inflammatory And Anti-Nociceptive Activities Of Methanolic Extract Of The Leaves Of Fraxinus floribunda Wallich. African Journal of Traditional, Complementary and Alternative Medicines. 2007;4: 411–416. doi:10.4314/ajtcam.v4i4.31235

10. Subba A, Sahu R, Bhardwaj S, Mandal P. Alpha Glucosidase Inhibiting Activity and in vivo Antidiabetic Activity of Fraxinus floribunda Bark in Streptozotocin‑Induced Diabetic Rats. Pharmacognosy Research. 2019;11: 273–278. doi:10.4103/pr.pr_32_19

11. Subba A, Dutta B, Sahu RK, Mandal P. Antioxidant, anti-inflammatory, and hepatoprotective activity of Fraxinus floribunda bark and the influence of extraction process on their bioactivity. Journal of Pharmacy Research. 2017;11.

12. Ghosh P, Ghosh C, Das S, Das C, Mandal S, Chatterjee S. Botanical Description, Phytochemical Constituents and Pharmacological Properties of Euphorbia hirta Linn: A Review. International Journal of Health Sciences. 2019.

13. Kumar S, Malhotra R, Kumar D. Euphorbia hirta: Its chemistry, traditional and medicinal uses, and pharmacological activities. Pharmacognosy Reviews. 2010;4: 58. doi:10.4103/0973-7847.65327

14. Sood SK, Bhardwaj R, Lakhanpal TN. Ethnic Indian Plants in Cure of Diabetes. Scientific Publishers; 2005.

15. Joseph N, Anjum N, Tripathi YC. Prunus cerasoides D. Don: A Review on Its Ethnomedicinal Uses, Phytochemistry and Pharmacology. International Journal of Pharmaceutical Sciences Review and Research. 2018;48(1: 62–69.

16. Kooltheat N, Tedasen A, Yamasaki K, Chatatikun M. Melanogenesis Inhibitory Activity, Chemical Components and Molecular Docking Studies of Prunus cerasoides Buch.-Ham. D. Don. Flowers. J Evid Based Complementary Altern Med. 2023;28: 2515690X231152928. doi:10.1177/2515690X231152928

17. Sachdeva C, Kumar S, Kaushik NK. Exploration of Anti-plasmodial Activity of Prunus cerasoides Buch.-Ham. ex D. Don (family: Rosaceae) and Its Wood Chromatographic Fractions. Acta Parasit. 2021;66: 205–212. doi:10.1007/s11686-020-00272-5

18. Sharma A, Joshi R, Kumar S, Sharma R, Rajneesh, Padwad Y, et al. Prunus cerasoides fruit extract ameliorates inflammatory stress by modulation of iNOS pathway and Th1/Th2 immune homeostasis in activated murine macrophages and lymphocytes. Inflammopharmacol. 2018;26: 1483–1495. doi:10.1007/s10787-018-0448-2

19. Afrin SR, Islam MR, Khanam BH, Proma NM, Didari SS, Jannat SW, et al. Phytochemical and pharmacological investigations of different extracts of leaves and stem barks of Macropanax dispermus (Araliaceae): a promising ethnomedicinal plant. Futur J Pharm Sci. 2021;7: 165. doi:10.1186/s43094-021-00313-4

20. Bisht A, Rajab B, Alghamdi S, Kamal M, Asif M. Study on In Vitro Antidiabetic Potential of Whole Plant Part of Urtica parviflora Roxb. LATIN AMERICAN JOURNAL OF PHARMACY. 2022;41: 1991–7.

21. Ozukum A, Changkija S, Tripath S. Ethnobotanical studies on the Khiamniungan tribe in Tuensang district of Nagaland, Northeast India: Ethnomedicinal plants. East Himalayan Society for Spermatophyte Taxonomy. 2019;13(01): 70–81.

22. Tamboli AM, Wadkar KA. A Recent Review on Phytochemical Constituents and Medicinal Properties of Mimosa rubicaulis Lam. IJSRST. 2019; 300–305. doi:10.32628/IJSRST196251

23. Genest S, Kerr C, Shah A, Rahman MM, Saif-E-Naser GMM, Nigam P, et al. Comparative bioactivity studies on two Mimosa species. 2008;7.

24. Gurung R, Adhikari S, Koirala N, Parajuli K. Extraction and Evaluation of Anti-inflammatory and Analgesic Activity of Mimosa rubicaulis in Swiss Albino Rats. Anti-Infective Agents. 2021;19: 30–37. doi:10.2174/2211352518999201009125006

25. Maneechai S, Likhitwitayawuid K, Sritularak B, Palanuvej C, Ruangrungsi N, Sirisa-ard P. Quantitative Analysis of Oxyresveratrol Content in Artocarpus lakoocha and ‘Puag-Haad.’ Medical Principles and Practice. 2009;18: 223–227. doi:10.1159/000204354

26. Chatsumpun N, Chuanasa T, Sritularak B, Lipipun V, Jongbunprasert V, Ruchirawat S, et al. Oxyresveratrol: Structural Modification and Evaluation of Biological Activities. Molecules. 2016;21: 489. doi:10.3390/molecules21040489

27. Jagtap UB, Bapat VA. *Artocarpus*: A review of its traditional uses, phytochemistry and pharmacology. Journal of Ethnopharmacology. 2010;129: 142–166. doi:10.1016/j.jep.2010.03.031

28. Sharan Shrestha S, Sut S, Ferrarese I, Barbon Di Marco S, Zengin G, De Franco M, et al. Himalayan Nettle Girardinia diversifolia as a Candidate Ingredient for Pharmaceutical and Nutraceutical Applications—Phytochemical Analysis and In Vitro Bioassays. Molecules. 2020;25: 1563. doi:10.3390/molecules25071563

29. Subedee BR, Chaudhary RP, Uprety Y, Dorji T. Socio-ecological perspectives of Himalayan Giant Nettle (Girardinia diversifolia (Link) Friis) in Nepal. Journal of Natural Fibers. 2020;17: 9–17. doi:10.1080/15440478.2018.1458684

30. Devi N. Medicinal Values of Trichosanthus cucumerina L. (Snake Gourd) - A Review. BJPR. 2017;16: 1–10. doi:10.9734/BJPR/2017/33575

31. Sari TA. Overview of Traditional Use, Phytochemical and Pharmacological Activities of Cucumber (Cucumis sativus L.). IJPSM. 2021;6: 39–49. doi:10.47760/ijpsm.2021.v06i03.004

32. Sharifi-Rad J, Quispe C, Turgumbayeva A, Mertdinç Z, Tütüncü S, Aydar EF, et al. Santalum Genus: phytochemical constituents, biological activities and health promoting-effects. Zeitschrift für Naturforschung C. 2023;78: 9–25. doi:10.1515/znc-2022-0076

33. Singh P, Dhankhar J, Kapoor RK, Sharma A. A comparative study on GC-MS analysis and antimicrobial activity of bioactive compounds present in aerial parts (leaf and fruit) of Ficus benghalensis L. Journal of Applied and Natural Science. 2023;15: 870–883. doi:10.31018/jans.v15i2.4618

34. VERMA V, Sehgal N, OmPrakash. CHARACTERIZATION AND SCREENING OF BIOACTIVE COMPOUNDS IN THE EXTRACT PREPARED FROM AERIAL ROOTS OF FICUS BENGHALENSIS. International Journal of Pharmaceutical Sciences and Research. 2015;6: 5059–5069. doi:10.13040/IJPSR.0975-8232.6(12).5056-69

35. Painuli S, Quispe C, Herrera-Bravo J, Semwal P, Martorell M, Almarhoon ZM, et al. Nutraceutical Profiling, Bioactive Composition, and Biological Applications of Lepidium sativum L. Oxidative Medicine and Cellular Longevity. 2022;2022: 2910411. doi:10.1155/2022/2910411

36. Alqahtani FY, Aleanizy FS, Mahmoud AZ, Farshori NN, Alfaraj R, Al-sheddi ES, et al. Chemical composition and antimicrobial, antioxidant, and anti-inflammatory activities of *Lepidium sativum* seed oil. Saudi Journal of Biological Sciences. 2019;26: 1089–1092. doi:10.1016/j.sjbs.2018.05.007

37. Gupta BD, Banerjee SK, Handa KL. Alkaloids and coumarins of Heracleum wallichii. Phytochemistry. 1976;15: 576. doi:10.1016/S0031-9422(00)88988-8

38. Bahadori MB, Dinparast L, Zengin G. The Genus Heracleum: A Comprehensive Review on Its Phytochemistry, Pharmacology, and Ethnobotanical Values as a Useful Herb. Comprehensive Reviews in Food Science and Food Safety. 2016;15: 1018–1039. doi:10.1111/1541-4337.12222

39. Bhattacharjee M, Y VS, Pratimsarma M. STUDY ON MULTIPOTENT MEDICINAL ASPECTS OF SCHIMA WALLICHII (BARK) FROM NAGALAND, NE INDIA. Asian Journal of Pharmaceutical and Clinical Research. 2019; 155–158. doi:10.22159/ajpcr.2019.v12i3.29288

40. Dewanjee S, Mandal V, Sahu R, Dua TK, Manna A, Mandal SC. Anti-inflammatory activity of a polyphenolic enriched extract of Schima wallichii bark. Natural Product Research. 2011;25: 696–703. doi:10.1080/14786410802560732

41. Lalhminghlui K, Jagetia GC. Evaluation of the anticancer activity of Chilauni, Schima wallichii (DC.) Korth. in vitro.

42. Wang C, Zhang Y, Ding H, Song M, Yin J, Yu H, et al. Authentication of Zingiber Species Based on Analysis of Metabolite Profiles. Front Plant Sci. 2021;12. doi:10.3389/fpls.2021.705446

43. Jamir K, Ganguly R, Seshagirirao K. ZCPG, a cysteine protease from *Zingiber montanum* rhizome exhibits enhanced anti-inflammatory and acetylcholinesterase inhibition potential. International Journal of Biological Macromolecules. 2020;163: 2429–2438. doi:10.1016/j.ijbiomac.2020.09.097

44. Paramita S, Aminyoto M, Ismail S, Arung ET. Anti-hypercholesterolemic effect of *Zingiber montanum* extract. F1000Research; 2019. doi:10.12688/f1000research.16417.2

45. Idris FN, Mohd Nadzir M. Comparative Studies on Different Extraction Methods of Centella asiatica and Extracts Bioactive Compounds Effects on Antimicrobial Activities. Antibiotics. 2021;10: 457. doi:10.3390/antibiotics10040457

46. Roy DC, Barman SK, Shaik MM. Current Updates on Centella asiatica : Phytochemistry, Pharmacology and Traditional Uses. Medicinal Plant Research. 2013;3. Available: https://hortherbpublisher.com/index.php/mpr/article/view/607

47. Sun B, Wu L, Wu Y, Zhang C, Qin L, Hayashi M, et al. Therapeutic Potential of Centella asiatica and Its Triterpenes: A Review. Front Pharmacol. 2020;11. doi:10.3389/fphar.2020.568032

48. Iqbal D, Khan MS, Khan MohdS, Ahmad S, Srivastava AK. An In Vitro and Molecular Informatics Study to Evaluate the Antioxidative and β-hydroxy-β-methylglutaryl-CoA Reductase Inhibitory Property of Ficus virens Ait. Phytotherapy Research. 2014;28: 899–908. doi:10.1002/ptr.5077

49. Chen X-X, Shi Y, Chai W-M, Feng H-L, Zhuang J-X, Chen Q-X. Condensed Tannins from Ficus virens as Tyrosinase Inhibitors: Structure, Inhibitory Activity and Molecular Mechanism. PLOS ONE. 2014;9: e91809. doi:10.1371/journal.pone.0091809

50. Chaturvedi M, Rani R, Sharma D, Yadav JP. Comparison of Curcuma Caesia extracts for bioactive metabolite composition, antioxidant and antimicrobial potential. Natural Product Research. 2021;35: 3131–3135. doi:10.1080/14786419.2019.1687472

51. Ibrahim NNA, Wan Mustapha WA, Sofian-Seng N-S, Lim SJ, Mohd Razali NS, Teh AH, et al. A Comprehensive Review with Future Prospects on the Medicinal Properties and Biological Activities of Curcuma caesia Roxb. Evidence-Based Complementary and Alternative Medicine. 2023;2023: 7006565. doi:10.1155/2023/7006565

52. Akhil BS, Ravi RP, Lekshmi A, Abeesh P, Guruvayoorappan C, Radhakrishnan KV, et al. Exploring the Phytochemical Profile and Biological Activities of Clerodendrum infortunatum. ACS Omega. 2023;8: 10383–10396. doi:10.1021/acsomega.2c08080

53. Luan F, Wu Q, Yang Y, Lv H, Liu D, Gan Z, et al. Traditional Uses, Chemical Constituents, Biological Properties, Clinical Settings, and Toxicities of Abelmoschus manihot L.: A Comprehensive Review. Front Pharmacol. 2020;11. doi:10.3389/fphar.2020.01068

54. Zhou Y, Li S-H, Jiang R-W, Cai M, Liu X, Ding L-S, et al. Quantitative analyses of indoloquinazoline alkaloids in Fructus Evodiae by high-performance liquid chromatography with atmospheric pressure chemical ionization tandem mass spectrometry. Rapid Communications in Mass Spectrometry. 2006;20: 3111–3118. doi:10.1002/rcm.2705

55. Pang Y-M, Shan Q, Zhou F-J, Hua J, Hou W-B. Chemical constituents and pharmacological effects of genus *Engelhardia*. Chinese Herbal Medicines. 2018;10: 2–13. doi:10.1016/j.chmed.2018.01.007

56. Sindhu R, Ashok Kumar U, Arora S. SANTALUM ALBUM LINN: A REVIEW ON MORPHOLOGY, PHYTOCHEMISTRY AND PHARMACOLOGICAL ASPECTS. International Journal of PharmTech Research. 2010;2(1): 914–919.

57. Fei Y, Wang J, Peng B, Peng J, Hu J-H, Zeng Z-P, et al. Phenolic constituents from Rheum nobile and their antioxidant activity. Natural Product Research. 2017;31: 2842–2849. doi:10.1080/14786419.2017.1303691

58. Subba AR, Rai SK, Gurung J, Singh B. An investigation on antioxidant and anti-arthritic activity of Rheum nobile Hook.f. & Thomson from Sikkim Himalayan region. Indian Journal of Natural Products and Resources (IJNPR) [Formerly Natural Product Radiance (NPR)]. 2023;14: 434–443. doi:10.56042/ijnpr.v14i3.4786

59. Arsyad AS, Nurrochmad A, Fakhrudin N. Phytochemistry, traditional uses, and pharmacological activities of Ficus elastica Roxb. ex Hornem: A review. J Herbmed Pharmacol. 2022;12: 41–53. doi:10.34172/jhp.2023.04

60. Ginting CN, Lister INE, Girsang E, Riastawati D, Kusuma HSW, Widowati W. Antioxidant Activities of Ficus elastica Leaves Ethanol Extract and Its Compounds. Molecular and Cellular Biomedical Sciences. 2020;4: 27–33. doi:10.21705/mcbs.v4i1.86

61. Chaniad P, Sudsai T, Septama AW, Chukaew A, Tewtrakul S. Evaluation of Anti-HIV-1 Integrase and Anti-Inflammatory Activities of Compounds from Betula alnoides Buch-Ham. Advances in Pharmacological and Pharmaceutical Sciences. 2019;2019: 2573965. doi:10.1155/2019/2573965

62. Ghimire BK, Tamang JP, Yu CY, Jung SJ, Chung IM. Antioxidant, antimicrobial activity and inhibition of α-glucosidase activity by Betula alnoides Buch. bark extract and their relationship with polyphenolic compounds concentration. Immunopharmacol Immunotoxicol. 2012;34: 824–831. doi:10.3109/08923973.2012.661739

63. Omar AM, Sun S, Kim MJ, Phan ND, Tawila AM, Awale S. Benzophenones from Betula alnoides with Antiausterity Activities against the PANC-1 Human Pancreatic Cancer Cell Line. J Nat Prod. 2021;84: 1607–1616. doi:10.1021/acs.jnatprod.1c00150
