# Supplementary material for: Network pharmacological insight into traditional bone healing practices of Sikkim, India
Source: PLoS One. 2026 Apr 15;21(4):e0346125. doi: 10.1371/journal.pone.0346125 (PMC13082723; doi:10.1371/journal.pone.0346125)
Supplement: Attachment — Submitted filename: Comments Plos One.docx [file pone.0346125.s025.docx]

**Network Pharmacological Insight into Traditional Bone Healing Practices of Sikkim, India (PONE-D-25-60628)**

The research is carefully executed and descriptive enough to strengthen the pharmacological knowledge of the traditionally used herbal formulation. The findings are thoughtfully integrated and offer promising therapeutic perspectives. Most importantly, such study will preserved valuable traditional healthcare knowledge of traditional healers. Moreover, all traditional healers participated in this study were included as author in this manuscript, which is very much necessary to acknowledge the credit of real knowledge holder.

**However, here are some of the suggestions that can help to improve the clarity and coherence of the manuscript**

- In Pathway validation for bone mending section, Eleven pathways (e.g., calcium signalling, RANKL/RANK, Vitamin D-sensitive Ca) are listed without enrichment statistics (p-values, fold-change) or hub gene metrics in the PPI network (TERT, SRC, CYP27B1). Provide quantitative enrichment data and explain how these specifically support calcium reabsorption in fracture repair.
- In Correlation of key Genes, from the top 10 genes the remarkable survival target genes AKT and STAT are common in cancer and other pathway. How does it correlate in bone mending?
- The English language is modest and contains several grammatical and typographical errors.
- Author may include detail methodology of documentation including questioners in supplementary file.

**Recommended for the publication with minor correction**
