# Supplementary material for: Network pharmacological insight into traditional bone healing practices of Sikkim, India
Source: PLoS One. 2026 Apr 15;21(4):e0346125. doi: 10.1371/journal.pone.0346125 (PMC13082723; doi:10.1371/journal.pone.0346125)
Supplement: Attachment — Submitted filename: R2 Response to reviewers 27-2-2026.docx [file pone.0346125.s027.docx]

**Response to Reviewers**

**Dear**

**Academic Editor
PLOS One**

**Re: – Response to Editor**

1. *Clearly define probability cut-offs, number of compounds failing Lipinski/Veber.*

**Response:** We sincerely thank the Editor for this insightful comment concerning the clarification of probability cut-off values and the number of compounds failing the Lipinski and Veber criteria. In response, we have revised the manuscript to clearly define the applied probability thresholds and to explicitly report the number of compounds that did not comply with the Lipinski and Veber rules, as suggested.

2. *Specify whether predicted targets relate to renal Ca²⁺ or osteoblast/osteoclast intracellular Ca²⁺ signaling.*

**Response:** We thank the editor for this valuable suggestion clarify whether the predicted targets are associated with renal Ca²⁺ regulation or intracellular Ca²⁺ signalling in osteoblasts and osteoclasts. In response, we have revised the manuscript to explicitly specify the physiological relevance of the predicted targets, clearly distinguishing those involved in renal Ca²⁺ handling from those participating in intracellular Ca²⁺ signalling pathways in osteoblasts and osteoclasts. These clarifications have been incorporated into the revised manuscript.

3. *Discussion should consistently reflect the exploratory and in silico nature of the findings.*

**Response:** We thank the reviewer for this important observation. In accordance with the comment, the Discussion section has been carefully revised to consistently emphasise the exploratory and in silico nature of the findings. Additionally, any statements implying experimental or clinical validation have been rephrased to clearly reflect that the results are based on computational predictions and require further experimental confirmation.

4. *Several species names remain misspelled. Ensure uniform italics and correct spelling throughout text, tables, and figures.*

**Response:** We thank the reviewer for drawing attention to this issue. The manuscript has been thoroughly reviewed, and all species names have been corrected for spelling accuracy and formatted consistently in italics throughout the text, tables, and figures, in accordance with standard taxonomic conventions.
