# Supplementary material for: Network pharmacological insight into traditional bone healing practices of Sikkim, India
Source: PLoS One. 2026 Apr 15;21(4):e0346125. doi: 10.1371/journal.pone.0346125 (PMC13082723; doi:10.1371/journal.pone.0346125)
Supplement: Attachment — Submitted filename: Reviewer comments.docx [file pone.0346125.s024.docx]

Network Pharmacological Insight into Traditional Bone Healing Practices of Sikkim, India, PONE-D-25-60628

**Strength of the Manuscript**

- An extensive cross culture ethno-pharmacological survey of an area of Sikkim Himalaya, which was less explored. This documentation work conserved valuable traditional knowledge of folklore practitioners.
- Traditional claims of folklore practitioners documented during the survey was validated using AI based network Pharmacological screening that preliminarily justified traditional claims for pharmacological uses of medicinal plants for treatment of bone fracture.

**Weakness of the Manuscript**

- **Phytochemical inclusion criteria**: From 1,043 compounds, 527 were selected after redundancy removal and ADMET screening, yet "clinical evidence" allowed retention of failures (e.g., high MW flavonoids). Specify exact ADMET parameters (Lipinski/Veber scores), violation rates, and which retained compounds link to calcium reabsorption pathways.
- **Calcium reabsorption**: Given that Calcium reabsorption is the critical step in bone mending, are there specific pathways anticipated in the calcium reabsorption from the network pharmacological annotations that need to addressed?
- **Scientific Name of Plant:** the scientific name of the all plants should be in italic.
- **Languages of Manuscript:** grammatical error and long sentences should me recheck for its correctness.
